# Phenotyping reproductive stage chilling and frost tolerance in wheat using targeted metabolome and lipidome profiling

**DOI:** 10.1007/s11306-019-1606-2

**Published:** 2019-10-20

**Authors:** Bo Eng Cheong, William Wing Ho Ho, Ben Biddulph, Xiaomei Wallace, Tina Rathjen, Thusitha W. T. Rupasinghe, Ute Roessner, Rudy Dolferus

**Affiliations:** 1 School of BioSciences, The University of Melbourne, Melbourne, VIC 3010 Australia; 2 CSIRO Agriculture & Food, GPO Box 1700, Canberra, ACT 2601 Australia; 3 Department of Primary Industries and Regional Development, 3 Baron Hay Court, South Perth, WA 6151 Australia; 4 Metabolomics Australia, School of BioSciences, The University of Melbourne, Melbourne, VIC 3010 Australia; 5 Melbourne Integrative Genomics, Schools of Mathematics and Statistics and of BioSciences, The University of Melbourne, Melbourne, VIC 3010 Australia

**Keywords:** Cold tolerance, Flowering, Lipid, Metabolite, Phenotyping, Wheat

## Abstract

**Introduction:**

Frost events lead to A$360 million of yield losses annually to the Australian wheat industry, making improvement of chilling and frost tolerance an important trait for breeding.

**Objectives:**

This study aimed to use metabolomics and lipidomics to explore genetic variation in acclimation potential to chilling and to identify metabolite markers for chilling tolerance in wheat.

**Methods:**

We established a controlled environment screening assay that is able to reproduce field rankings of wheat germplasm for chilling and frost tolerance. This assay, together with targeted metabolomics and lipidomics approaches, were used to compare metabolite and lipid levels in flag leaves of two wheat varieties with contrasting chilling tolerance.

**Results:**

The sensitive variety Wyalkatchem showed a strong reduction in amino acids after the first cold night, followed by accumulation of osmolytes such as fructose, glucose, putrescine and shikimate over a 4-day period. Accumulation of osmolytes is indicative of acclimation to water stress in Wyalkatchem. This response was not observed for tolerant variety Young. The two varieties also displayed significant differences in lipid accumulation. Variation in two lipid clusters, resulted in a higher unsaturated to saturated lipid ratio in Young after 4 days cold treatment and the lipids PC(34:0), PC(34:1), PC(35:1), PC(38:3), and PI(36:4) were the main contributors to the unsaturated to saturated ratio change. This indicates that Young may have superior ability to maintain membrane fluidity following cold exposure, thereby avoiding membrane damage and water stress observed for Wyalkatchem.

**Conclusion:**

Our study suggests that metabolomics and lipidomics markers could be used as an alternative phenotyping method to discriminate wheat varieties with differences in cold acclimation.

**Electronic supplementary material:**

The online version of this article (10.1007/s11306-019-1606-2) contains supplementary material, which is available to authorized users.

## Introduction

For important food crops, such as cereals, chilling and frost are major constraints to yield and productivity (Gray et al. 1997; Leff et al. 2004; Lobell and Gourdji 2012). Climate change affects atmospheric conditions, increasing the probability of weather extremes. This is likely to result in more frequent crop losses in the future (Lobell and Gourdji 2012). Warmer winters and more frequent spring frosts have increasingly affected wheat yields in China between 1961 and 2000 (Li et al. 2014, 2015). In Southern Australia, spring frosts have become significantly more frequent since 1960 and the overall length of the frost season has increased by 1 month, resulting in more frequent occurrences of frost damage to wheat crops (Crimp et al. 2015; Zheng et al. 2015). In Australia, spring wheat varieties are sown in autumn, flower in early spring and are harvested in late spring (Zheng et al. 2015). Radiant frosts in spring are caused by heat loss from the soil surface to the night sky on cold and dry days with clear skies. This leads to a rapid cooling of the crop canopy and associated frost damage if crops are at susceptible stages (Marcellos and Single 1975). The associated increase in risk of frost damage requires wheat cultivars with improved frost tolerance. Reoccurring frost events cost the Australian wheat industry an estimated A$360 million of direct and indirect yield losses annually (March et al. 2015; Zheng et al. 2015).

Above zero degree temperatures (chilling; typically 10 to 0 °C) cause an acclimation or adaptation response in plant tissues. Below zero degree temperatures (frosts) cause an additional challenge to plant tissues due to the formation of ice crystals. Quantitative and/or qualitative differences in the acclimation response during the chilling period may provide various degrees of frost tolerance. Wheat tolerates frosts during vegetative growth, but becomes more frost-sensitive during flowering in early spring. Chilling conditions during flowering can cause pollen sterility and grain loss in wheat (Demotes-Mainard et al. 1996; Rerkasem 1996; Subedi et al. 1998a, b, 2000; Chakrabarti et al. 2011). Exposure of plants to chilling conditions (“hardening”) enhances freezing tolerance in a process called cold acclimation (Palta and Weiss 1993; Guy 1999; Tomashow 1999; Wanner and Junttila 1999). In wheat, cold acclimation during reproductive stages remains poorly characterised. Although most genes involved in cold acclimation are expressed after 24 h of exposure (Monroy et al. 2007; Kurepin et al. 2013), the full establishment of the response may require prolonged or repeated exposures to cold (Levitt 1980; Guy 1990; Ruelland et al. 2009). Cold acclimation can be lost during warmer fluctuating winter conditions, but it remains unclear whether re-acclimation can occur following de-acclimation (Rapacz 2002; Kalberer et al. 2006; Vitamvas and Prasil 2008), particularly after crops transition into reproductive stages. Loss of cold acclimation during spring makes wheat crops particularly vulnerable to unexpected frosts (Frederiks et al. 2011, 2012). Cold temperatures, in combination with increasing day-length, accelerate the transition from vegetative to reproductive growth as the vernalization and photoperiod requirements are met (Fowler et al. 2001; Limin and Fowler 2006). Proper management of sowing time and phenology of wheat (flowering time, response to day-length) minimise frost damage, but the unpredictability of frost events requires genetic improvement in frost tolerance to manage yield stability (Limin and Fowler 2006; Zheng et al. 2015), particulary in Southern Australia where the length of the frost season is increasing (Crimp et al. 2015) and crop development can not be delayed further.

Induction of cold acclimation and freezing tolerance involves physiological and biochemical changes in plants (Wanner and Junttila 1999). Cold-tolerant plants have a higher proportion of unsaturated fatty acids in the plasma membrane and are better at maintaining membrane fluidity at lower temperatures (Vigh et al. 1979; Steponkus et al. 1993; Bohn et al. 2007). Plants tend to increase the degree of fatty acid unsaturation and the content of phospholipids when they are exposed to low, non-freezing temperatures (Welti et al. 2007). In Arabidopsis, phophatidylcholines (PC), phosphatidylethanolamines (PE), phosphatidylglycerols (PG) and monogalactosyldiacylglycerols (MGDG) containing two polyunsaturated acyl species, including 36:5 (18:2/18:3) and 36:6 (di-18:3), increased during cold acclimation (Welti et al. 2002). Chilling conditions also lead to the accumulation of osmolytes, compatible solutes or cryoprotectants such as sugars (fructose, glucose, trehalose, raffinose) and amino acids or amines such as proline and gamma-aminobutyric acid (GABA). This response is not specific to chilling and is also induced by other stressors that cause osmotic stress (e.g., drought, salinity, heat; Beck et al. 2007). Apart from playing a role in compensating for water loss during osmotic adjustment, osmolytes are also important for protecting membrane integrity and scavenging of reactive oxygen species (Guy 1999; Wanner and Junttila 1999; Smallwood and Bowles 2002; Valluru et al. 2008; Javadian et al. 2010; Verslues et al. 2006; Janmohammadi 2012). Cold acclimation in spring and winter *Triticum monococcum* lines affects the steady-state levels of phytohormones, including abscisic acid (ABA), salicylic acid, ethylene, jasmonic acid, gibberellins, cytokinins and auxin (Horvath et al. 2007; Machakova et al. 1989; Vanková et al. 2014). Increased ABA levels are correlated with cold-induced water deficit in plants. ABA is implicated in plasma membrane lipid alterations during cold stress (Bohn et al. 2007). ABA induces the expression of phospholipase D (AtPLD) in Arabidopsis, causing hydrolysis of structural phospholipids (Zhang et al. 2005; Meijer and Munnik 2003).

Studying quantitative and qualitative differences in metabolite and lipid accumulation during cold acclimation is an important tool to understand chilling and frost tolerance in wheat. Targeted metabolomics and lipidomics using gas/liquid chromatography, coupled to a triple-quadruple MS (GC/LC-QqQ-MS) approach offers higher sensitivity, selectivity, reproducibility and robust quantification over a broad dynamic range (Douglas 2009; Sumner et al. 2015; Jorge et al. 2016). Dias et al. (2015) were able to quantify 76 primary metabolites (sugars, organic acids and amino acids/amines) in two chickpea cultivars with contrasting responses to salinity using GC- and LC-QqQ-MS. Natera et al. (2016) reported the quantification of 63 phospholipids in the roots of two barley genotypes with contrasting responses to salinity using LC-QqQ-MS. We therefore applied these approaches to study the cold acclimation response in wheat.

Reliable phenotyping for chilling and frost tolerance in the field is challenging due to unpredictability, variability in severity, duration, and timing of these events. In this study we established a reliable controlled environment (CE) phenotyping method to discriminate genetic variation in cold and frost tolerance in wheat. This CE phenotyping method, together with targeted metabolomics and lipidomics approaches, were then used to identify quantitative and qualitative differences in metabolite and lipid accumulation in two wheat varieties with differential chilling-tolerance. As starting tissue we used flag leaves from wheat plants harvested at the young microspore (YM) stage of pollen development, the reproductive stage with highest sensitivity to various abiotic stresses -including cold (Dolferus et al. 2013). The results indicate that two Australian spring wheat varieties with contrasting chilling tolerance (cold-tolerant Young and cold-sensitive Wyalkatchem) differed significantly in the accumulation of metabolites (amino acids, osmolytes) and levels of unsaturated versus saturated membrane lipids, indicating that metabolite profiling could be used to select wheat varieties with differences in cold acclimation.

## Materials and methods

### Controlled environment wheat growing, cold treatment and sample collection

Wheat varieties Wyalkatchem (cold-sensitive) and Young (cold-tolerant) used in this study were obtained via the National Frost Initiative, Australia (http://www.nvtonline.com.au/frost/). Seeds were sown in trays (36 × 25 × 13 cm, L × W × H; 15 plants/tray) filled with soil (100% composted soil, containing 1 g l^−1^ fertilizer: 14.4% N/6.6% P/5% K). Plants were grown in the glasshouse under natural lighting conditions and controlled temperature regime (24/16 °C, L/D); watering occurred once daily. The young microspore (YM) stage of pollen development was determined using auricle distance measurements (Ji et al. 2010; Dolferus et al. 2013). The Zadoks scale was used to determine growth stages other than YM (Zadoks et al. 1974). Cold treatments were carried out at the YM stage in a Conviron PGC 20 growth chamber. The treatment cycle consisted of 12 h incubation at 21 °C in the light, followed by a linear cooling gradient descending from 21 to − 3 °C over a period of 4 h, then followed by a continuous cold period at − 3 °C over 8 h in the dark (12/12 light/dark cycle, using 400 μmol m^−2^ s^−1^ light intensity; see Fig. S1a). The whole experiment was run over four consecutive days and samples were harvested in the morning immediately after a cold treatment at day one (TP_1_—short cold stress), day four (TP_3_—prolonged cold stress), and corresponding day-time samples were harvested 6 h into the normal temperature (21 °C) light cycle after day one and day four respectively (TP_2_ and TP_4_; see Fig. S1f). One tray of plants was used as untreated control to harvest T_0_ samples. In the trays for stress treatments, tillers that reached the YM stage were tagged before cold-treatment. Three flag leaves were harvested from three different tagged plants at each of the time points (TP_0_ to TP_4_) and pooled as one replicate for metabolite measurements. For each time point, we harvested four biological replicates per variety (n = 4) for all the metabolite and lipid analyses, except for phytohormone analyses where three replicates were used (n = 3). For sterility measurements, plants were returned to the glasshouse after cold treatment, and spike grain number of tagged tillers was determined at maturity.

### Analysis of sugars, organic acids, amino acids, amines and phytohormones

All chemicals and solvents for metabolite measurements were purchased from Sigma-Aldrich (Australia) and were of analytical or mass spectrometry grades. For sugars, organic acids and amines, tissue extraction was performed according to Dias et al. (2015), with some modifications. Aliquots of frozen leaf material (50 mg per replicate × four replicates) were weighed into Cryomill tubes (Precellys 24, Bertin Technologies). Subsequently, 500 µl of methanol containing 4% of internal standard (from a stock solution containing 0.5 mg ml^−1^ of ^12^C_6_-sorbitol and 0.5 mg ml^−1^ ^13^C_5_-^15^N-l-valine) was added to the samples, followed by vortexing for 30 s and homogenization at − 10 °C using a Cryomill (3 × 45 s at 6100 rpm). The samples were then extracted for 15 min at 30 °C in a thermomixer at 850 rpm, and subsequently centrifuged for 5 min at 4 °C at 13,000 rpm. The supernatants were transferred into new tubes, and 500 µl of water containing 0.2% formic acid was added into the Cryomill tubes containing the previously ground tissue pellet. The samples were vortex-mixed for 30 s, and centrifuged at 13,000 rpm for 5 min at 4 °C. The supernatants were then transferred and combined with the methanolic extracts from the previous centrifugation. A 200 µl of dichloromethane was added to the combined supernatants to separate chlorophylls. The combined supernatants were vortexed, centrifuged at 13,000 rpm for 2 min at 4 °C. The supernatants were taken and stored at − 80 °C for subsequent sugars, organic acids and amine analyses.

For the analysis of sugars and organic acids, 5 µl and 125 µl aliquots of the supernatants was transferred into new glass vial inserts, and dried *in vacuo* for sugars and organic acids analyses using GC-QqQ-MS. Prior to the GC-QqQ-MS analysis, the dried extracts were derivatized with methoxyamine hydrochloride in pyridine and bis-(trimethylsilyl)-trifluoroacetamide (BFTFA) as described by Dias et al. (2015). Briefly, All samples were re-dissolved in 20 µl of 30 mg ml^−1^ methoxyamine hydrochloride in pyridine and derivatized at 37 °C for 120 min with mixing at 500 rpm. The samples were incubated for 30 min with mixing at 500 rpm after addition of 20 µl *N*,*O*-bis-(trimethylsilyl)-trifluoroacetamide (BSTFA). Each derivatized sample was allowed to rest for 60 min prior to injection. Later, the derivatized samples (injection volume of 1 µl for each sample) were injected into a GC-QqQ-MS system comprising of a Gerstel 2.5.2 Autosampler, a 7890A Agilent gas chromatograph and a 7000 Agilent triple-quadruple MS (Agilent Santa Clara, USA) with an electron impact (EI) ion source. The instrument settings were the same as described by Dias et al. (2015). For calibration and quantification of sugars and organic acids in the flag leaf samples, we used serial concentrations of calibration authentic standards (Table S1), including 24 sugars (sugars, sugar phosphates, sugar acids and sugar alcohols) and 19 organic acids, derivatized and subjected to GC-QqQ-MS analysis–as described by Dias et al. (2015).

For the amino acid and amine analyses with LC-QqQ-MS, 10 µl of supernatants were transferred into new glass vial inserts. The amino acids and amines in the supernatants were then derivatized with 6-aminoquinolyl-*N*-hydrosysuccinimidyl carbamate (AQC) reagent as described in Boughton et al. (2011) and Dias et al. (2015). Briefly, 10 µl samples were added to 70 µl of borate buffer (200 mM, pH 8.8 at 25 °C) containing 10 mM TCEP, 1 mM ascorbic acid and 50 µM 2-aminobutyrate. The resulting solution was vortexed before adding 20 µl of 6-aminoquinolyl-*N*-hydrosysuccinimidyl carbamate (AQC) reagent [200 mM dissolved in 100% acetonitrile (ACN)] and then immediately vortexed. The samples were heated with shaking at 55 °C for 10 min, then centrifuged at 13,000 rpm at room temperature and transferred to HPLC vials containing inserts (Agilent, springless glass inserts, 250 µl) prior to injection. The derivatized samples (injection volume of 1 µl for each sample) were immediately injected into a LC-QqQ-MS system comprising of an Agilent 1200 LC-system coupled to an Agilent 6410 Electrospray Ionization-Triple Quadruple-MS. The setting of the LC–MS instrument were as described by Dias et al. (2015). For calibration and quantification of amino acids and amines in the flag leaf samples, a series of concentrations of calibration authentic standards (Table S1), comprising of 29 amino acids and amines mixed with sulfur-containing compound solution were prepared, derivatized and subjected to LC-QqQ-MS analysis same as described by Dias et al. (2015).

Seven phytohormones and phytohormone-related compounds [salicylic acid (SA), jasmonic acid (JA), jasmonoyl-isoleucine (JA-ile), indole-3-acetic acid (IAA), indole-3-carboxylic acid (ICA), indole-3-butyric acid (IBA) and 2-cis-4-trans-abscisic acid (ABA)], as well as three organic acids which are precursors for phytohormones [(benzoic acid (BA), trans-cinnamic acid (CA) and 12-oxo-phytodienoic acid (OPDA) were analysed in this study. Extraction for phytohormone analysis was carried out using a slightly modified procedure of Cao et al. (2017). Briefly, the flag leaf samples were ground with pestle and mortar in liquid nitrogen and frozen immediately. A 100 mg of sample was weighed for each of the samples and transferred to a dried 2 ml centrifuge tube that was pre-washed with MS grade methanol. 1 ml of extraction solvent, containing 956 µl of 70% methanol and 44 µl of 8.3 µg ml^−1^ internal standard mixtures (d_2_IAA, d_5_BA, d_6_ABA, d_6_SA, d_7_CA, H_2_JA), was added to the sample, then vortexed and agitated at 4 °C, and spun at 1400 rpm for 30 min. The samples were then centrifuged at 16,100×*g* for 10 min. The supernatants were transferred to fresh centrifuge tubes. The residues were re-extracted and centrifuged with 500 µl of extraction solvents (without internal standards). Finally, the first and second supernatant was combined and kept at − 80 °C until further derivatization steps using methyl-chloroformate (MCF). The subsequent derivatization steps were carried out according to Rawlinson et al. (2015), with some modifications. While Rawlinson et al. used GC coupled with single MS, our analysis used triple quadruple GC–MS (GC-QqQ-MS) with newly developed multiple reaction monitorings (MRMs) which were developed for each of the measured phytohormone and organic acids. Briefly, 600 µl of the supernatant for each sample was taken, dried down and re-dissolved with 160 µl methanol. Then, 8.3 µg ml^−1^ of ^13^C_5_-^15^N-l-Valine (another internal standard) was added. 34 µl of pyridine was then added to the mixture, followed by vortexing vigorously for 25–30 s. 200 µl of 1% NaOH solution was then added to the mixture. This ~ 400 µl mixture was then derivatized with 20 µl of methyl chloroformate (MCF) and vortexed vigorously for 25–30 s. Another 20 µl of methyl chloroformate (MCF) was added, followed by vortexing. 400 µl of chloroform was then added to the mixture, vortexed again for 10 s, followed by an addition of 400 µl of 50 mM sodium bicarbonate. The mixture was vortexed for 10–15 s and centrifuged for 30 s at 16,100×*g*. The upper aqueous layer was discarded and the bottom organic layer was taken (1 µl for each sample) and injected into a GC-QqQ-MS system comprising a Gerstel 2.5.2 Autosampler, a 7890A Agilent gas chromatograph and a 7010 Agilent triple-quadrupole MS (Agilent, Santa Clara, USA) with an electron impact (EI) ion source. More details for the preparation of calibration and internal standards, extraction, derivatization steps and GC-QqQ-MS instrument settings can be found in Supplementary Data S1 (Methodology for phytohormone analysis).

### Analysis of lipid content

The extraction of lipids was carried according to Folch et al. (1957), with modifications. Briefly, 30 mg freshly frozen flag leaf samples were homogenized in 500 µl of a 2:1 methanol:chloroform mixture using a Cryomill (Precellys 24, Bertin technologies) for 3 × 45 s at 6100 rpm (− 10 °C). The extracts were shaken at 750 rpm for 15 min at 30 °C, and centrifuged at 13,000 rpm for 15 min. The supernatants were transferred to new tubes, while 500 µl of 2:1 methanol:chloroform mixture was added again to each of the remaining residue, vortexed and centrifuged at 13,000 rpm for 15 min. The supernatants were combined with the previous supernatants. The combined supernatants were dried down *in vacuo* and re-constituted in 100 µl of 1:1 butanol:methanol for the subsequent LC-QqQ-MS analysis using an Agilent 1200 LC-system coupled to an Agilent 6410 Electrospray Ionization-Triple Quadruple-MS system. An injection of 5 µl of each total lipid extract was chromatographically separated on an Ascentis Express RP-Amide 50 × 2.1 mm, 2.7 µm HPLC column (Sigma-Aldrich, Castle Hill, NSW) using an 8 min gradient from 0% A to 100% B, which was then held for 2 min and followed by a 4 min column re-equilibration with a flow rate of 0.18 ml min^−1^. The mobile phases were: A, 10 mM ammonium formate in water: methanol: tetrahydrofuran (50: 20: 30, v/v/v); B, 10 mM ammonium formate in water: methanol: tetrahydrofuran (5: 20: 75, v/v/v). Lipid species were identified and quantified based on multiple reaction monitorings (MRMs) established using external lipid standards and references from the LIPIDMATCH database (https://github.com/GarrettLab-UF/LipidMatch/releases/tag/v2.0.2) as listed in Table S2, with a 5–20 ms dwell time for the simultaneous measurements of up to 100 compounds. We used optimised parameters for capillary (4000 V), fragmentor (60–160 V) and collision voltages (20–40 V). In all cases, the collision gas was nitrogen with a flow rate of 7 l min^−1^. The external lipid standards used (Table S2) were lysophosphatidylcholine LPC(17:0), phospatidylcholine PC(34:1), phosphatidylethanolamine PE(34:0), phosphatidylglycerol PG(34:1), phosphatidylinositol PI(36:2), phosphatidic acid (PA) and phosphatidylserine PS(34:0). Detected lipid species are annotated as follows: lipid class designation (total number of carbon atoms in the fatty acid chains: total number of double bonds in the fatty acid chains).

### Data processing and statistical analysis of metabolite data

**Fig. 1:**
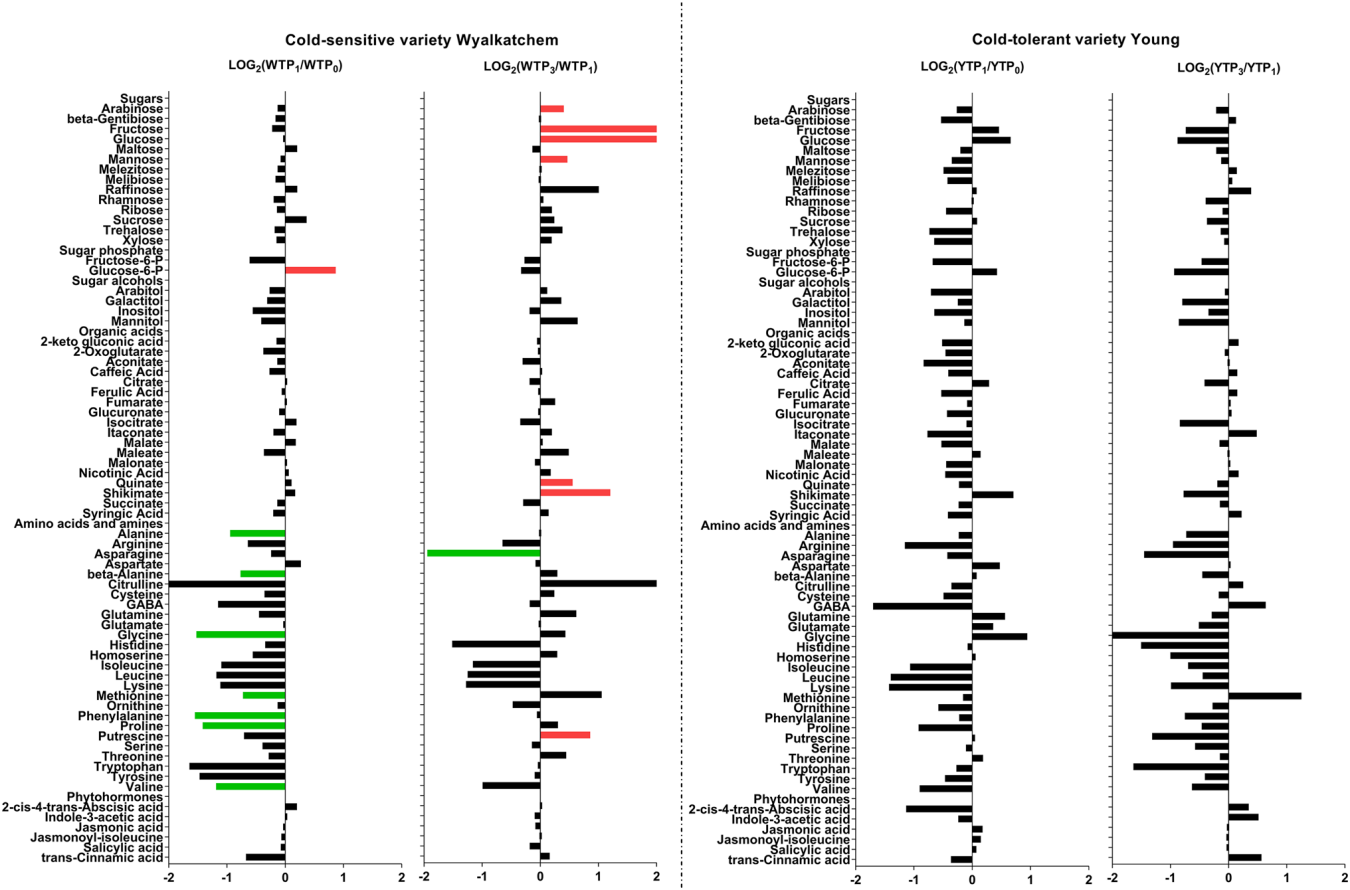
Log_2_-fold changes of primary metabolites in the flag leaves of the cold-sensitive Wyalkatchem (W) and cold-tolerant Young (Y) after one night (TP_1_ vs. TP_0_) and prolonged (TP_3_ vs. TP_1_) of cold treatment. Fold changes were calculated by dividing the concentration of the variety at a time point (e.g., TP_1_) to the concentration of that variety at the previous time point (e.g., TP_0_), then Log_2_-transformed. Significance of difference was determined by Benjamini and Hochberg method (Benjamini and Hochberg 1995) with false discovery rate (FDR)-adjusted *p*-value of 0.05 as the cut-off. *Green* significant decrease, *Red* significant increase. There were four biological replicates (n = 4) for all the measured metabolites, except phytohormones (n = 3)

**Fig. 2:**
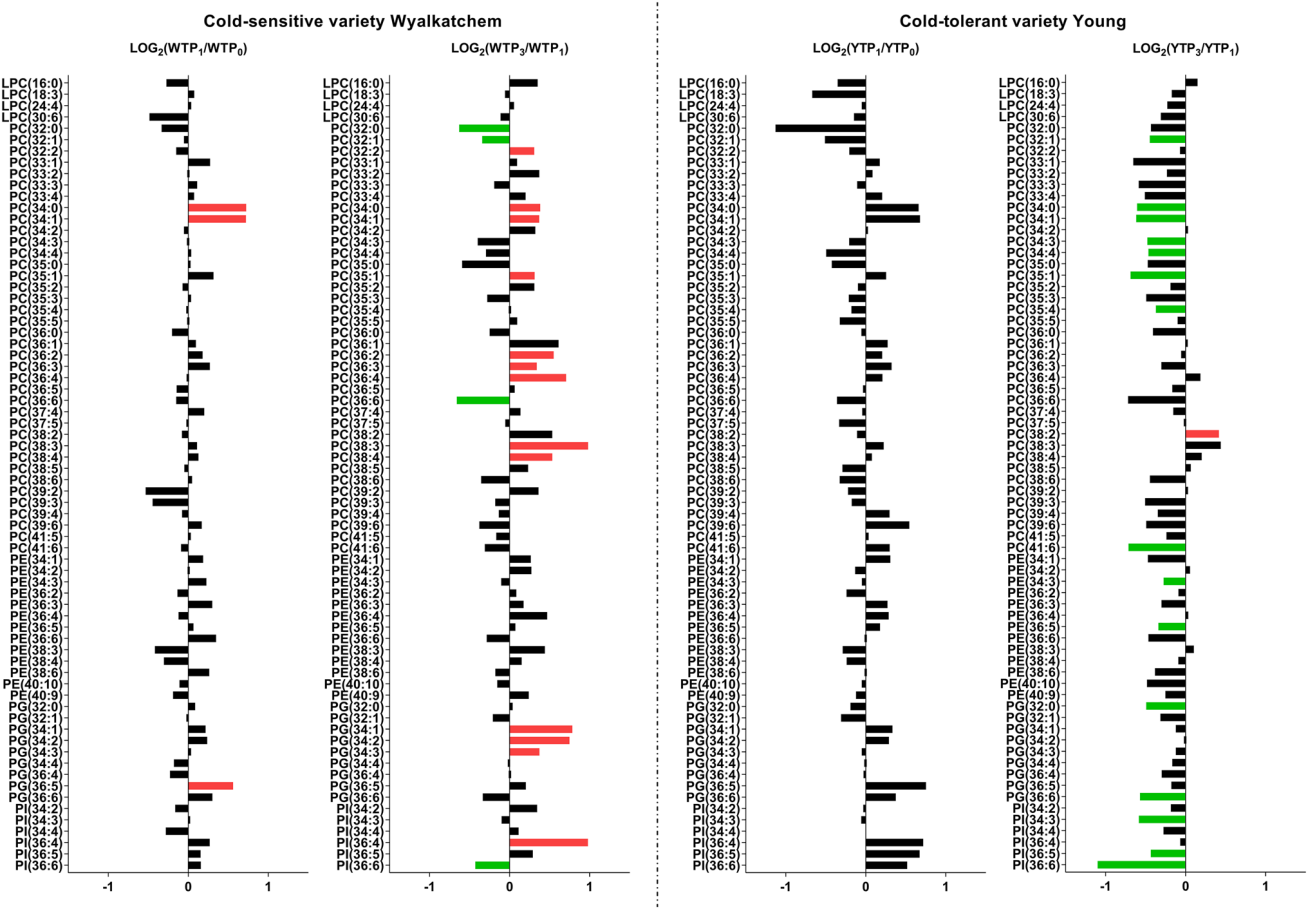
Log_2_-fold changes of phospholipids in the flag leaves of the cold-sensitive Wyalkatchem (W) and cold-tolerant Young (Y) after one night (TP_1_ vs. TP_0_) and prolonged (TP_3_ vs. TP_1_) of cold treatment. Fold changes were calculated by dividing the normalized response of the variety at a time point (e.g., TP_1_) to the normalized response of that variety at the previous time point (e.g., TP_0_), then Log_2_-transformed. Statistical method and cut-offs are as stated in Fig. 1. *Green* significant decrease, *Red* significant increase. There were four biological replicates (n = 4) for all the measured lipids

**Fig. 3:**
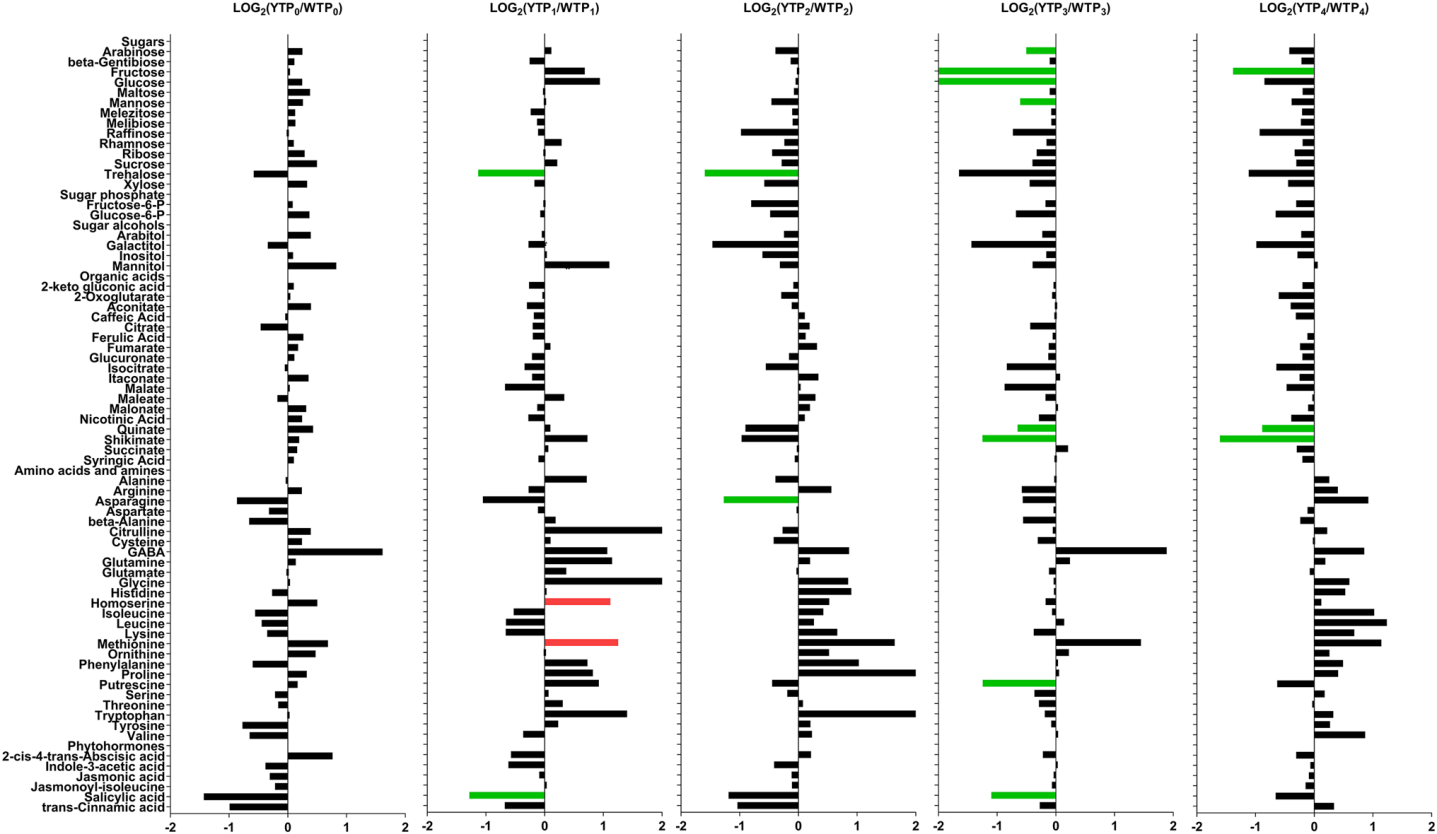
Log_2_-fold changes of primary metabolites in the flag leaves of cold-tolerant Young (Y) compared to the cold-sensitive Wyalkatchem (W) at each time point. Fold changes were calculated by dividing the concentration of Y to the concentration of W at that particular time point, then Log_2_-transformed. Statistical method and cut-offs are as stated in Fig. 1. *Green* significantly lower in Young/higher in Wyalkatchem; *Red* significantly higher in Young/lower in Wyalkatchem. There were four biological replicates (n = 4) for all the measured primary metabolites, except phytohormones (n = 3)

**Fig. 4:**
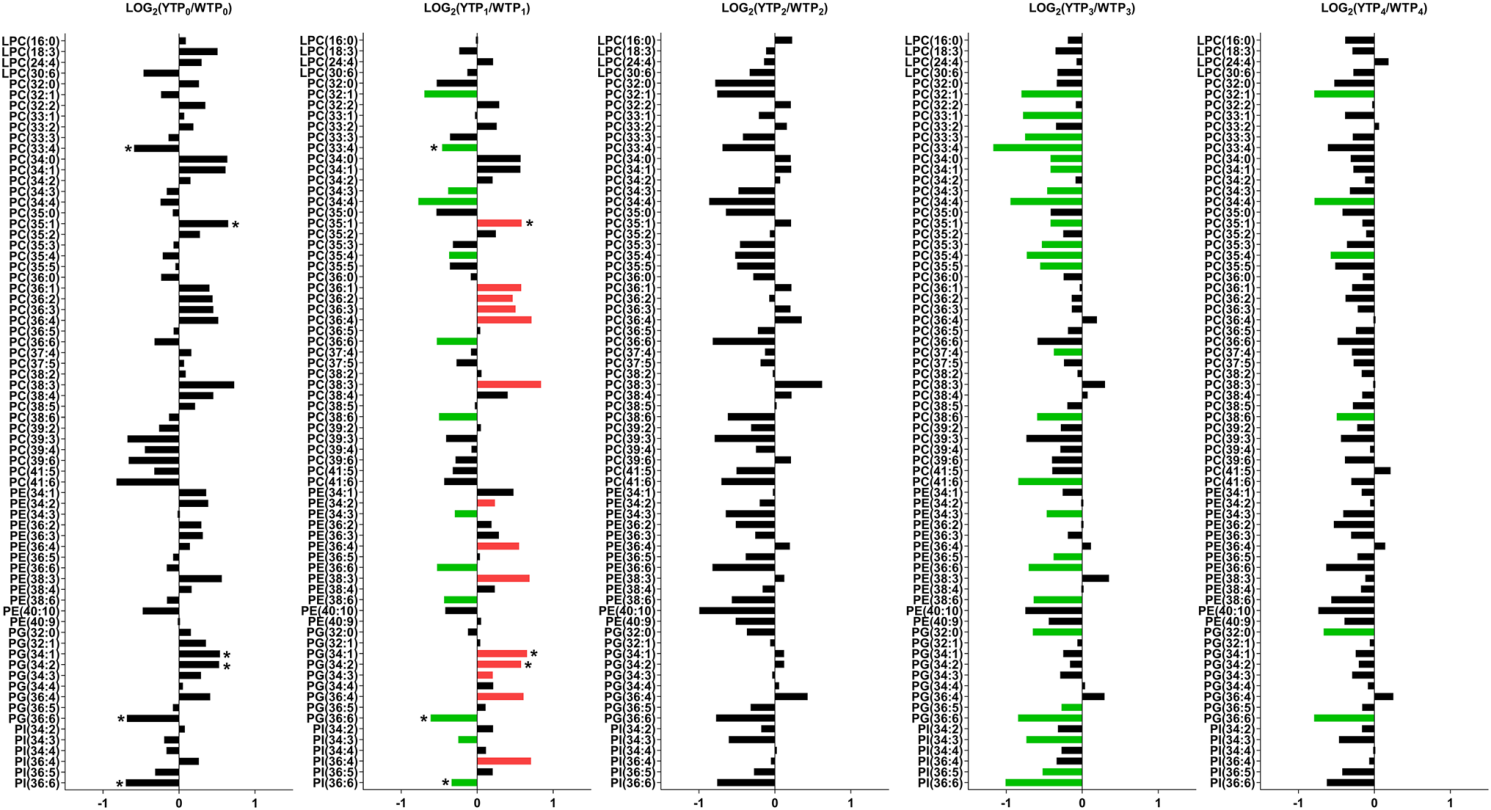
Log_2_-fold changes of phospholipids in the flag leaves of the cold-tolerant Young (Y) compared to the sensitive Wyalkatchem (W) at each time point. Fold changes were calculated by dividing the concentration of Y to the concentration of W at that particular time point, then Log_2_-transformed. Statistical method and cut-offs are as stated in Fig. 1. *Green* significantly lower in Young/higher in Wyalkatchem; *Red* significantly higher in Young/lower in Wyalkatchem. There were four biological replicates (n = 4) for all the measured lipids. Three lipids: PC(33:4), PG(36:6) and PI(36:6) were lower in Young compared to Wyalkatchem, while another three lipids: PC(35:1), PG(34:1) and PG(34:2) were higher in Young compared to Wyalkatchem when a cut-off of 0.1 was used. Although these six lipids (indicated with asterisks at TP_0_ and TP_1_) did not satisfy the significance threshold (*p *< 0.05) at TP_0_, they did become significantly different after the first night of cold treatment (TP_1_)

Raw metabolite and lipid data were processed and analyzed using Quantitative Analysis MassHunter Workstation software for QQQ (Agilent Technologies, Santa Clara, CA, USA). The level of identification was carried out based on the Metabolomics Standards Initiative (MSI) requirements (Sumner et al. 2007). For all measured metabolites (sugars, organic acids, amines and phytohormones), absolute quantities were determined while the concentration unit was expressed as picomole/mg of fresh weight (Table S3), using MSI Level 1 as the identification was based on Multiple Reaction Monitorings (MRMs) established using authentic standards (Table S1). Meanwhile, for lipids, responses were normalized to mg fresh weight (Table S4). Although single authentic lipid species were used for each of the phospholipid class, the identification of individual lipid species was based on the MRM experiment and retention time (MSI Level 2, Table S2). The processed metabolite and lipid data were first subjected to multiple comparison statistical analyses using Analysis of Variance (ANOVA), with a false discovery rate (FDR)-adjusted *p* value of 0.05 and using the Benjamini–Hochberg method (1995) to determine the metabolites and lipids that changed significantly across all time points or over the 4-day cold stress period, followed by the Tukey’s honestly significant difference (HSD) post hoc test in order to determine which pair-wise combinations showed significant differences for both primary metabolites and lipids using Graphpad Prism 7.0 (GraphPad Software, La Jolla California USA). Next, we determined which metabolites/lipids responded specifically to short cold stress (TP_1_), prolonged cold stress (TP_3_), diurnal fluctuations when the plants were exposed to normal day-time temperature of 21 °C for 6 h (TP_2_ and TP_4_). We therefore performed pairwise comparisons of the selected two groups TP_1_ versus TP_0_ (for short cold stress), TP_3_ versus TP_1_ (for prolonged cold stress), TP_2_ versus TP_1_ and TP_4_ versus TP_3_ (for recovery and the effect of diurnal fluctuations on metabolite levels) within each variety, and comparisons in every time point between the two varieties (varietal differences). The comparisons for metabolites were presented as Log_2_-transformed of fold change values (Figs. 1, 3; Table S5). A similar approach was also performed for the lipid data (Figs. 2, 4; Table S6). Statistical significance of differences observed between samples was evaluated with the Student’s *t*-test in Excel, with a false discovery rate (FDR)-adjusted *p*-value of 0.05 as the cut-off (Benjamini and Hochberg 1995). The statistical analysis for the comparison of the unsaturation to saturation ratio between Wyalkatchem and Young at different time points was also performed with Student’s *t*-test using GraphPad Prism 7.0 software (GraphPad Software, La Jolla, CA, USA). All bar plots were created using the same software.

## Results

### Establishment of a controlled environment assay for chilling tolerance

To establish a controlled environment phenotyping method for chilling and frost tolerance in wheat, we used two wheat varieties that consistently performed better (Young) and worse (Wyalkatchem) in terms of grain yield in the Australian National Frost Initiative (NFI) field trials. We used a linear cooling gradient descending from 21 to − 3 °C over a period of 4 h, followed by a continuous cold period at − 3 °C over 8 h (Fig. S1a). A four-day chilling treatment at the YM stage showed that the average spike grain number relative to unstressed control plants was consistently higher for Young compared to Wyalkatchem, with Wyalkatchem grain number reduced to an average of 76.3% of control (Fig. S1b). Reduced spike grain number in Wyalkatchem was often associated with the development of larger grains (Fig. S1c). A 4-day cold treatment during different stages of reproductive development showed that sensitivity to chilling in Wyalkatchem was highest at Zadok stages 41 to 47 and from 57 to 65, which corresponded to the YM stage and anthesis respectively. Spike grain numbers in Young were not significantly affected at both stages (Fig. S1d). A time course experiment shows that spike grain numbers in Wyalkatchem were reduced considerably after three days of treatment, while in Young spike grain numbers were reduced significantly from 5 days treatment onwards (Fig. S1e). We therefore standardised on a 4-day YM stage treatment to discriminate the two varieties.

### Four-day chilling treatment induces significant changes in metabolites and lipids in Young and Wyalkatchem

To develop a workable field phenotyping method, we focused on cold-induced metabolite changes in the flag leaf, a tissue that is easier to collect in a non-destructive way compared to YM stage spikes. In the field, wheat plants often experience consecutive nights of chilling and frost events throughout flowering, as frost events are linked to large scale climatic patterns and regularly occur over successive nights, while day time temperatures can be normal (~ 20 °C). Previous chilling events can influence metabolite levels at any harvest time. A time course experiment was therefore designed to identify metabolite changes that were stable over consecutive cold events and were not subject to day-time recovery or to circadian fluctuations. In addition, metabolite markers for chilling tolerance have to accumulate to significantly different levels in the cold-tolerant and sensitive wheat lines.

Using ANOVA multiple comparison analysis and a false discovery rate (FDR)-adjusted *p*-value of 0.05, significant changes of metabolites and lipids were observed in each variety and between the two varieties across all the time points (Table 1, S7, S8). Cold treatment of cold-sensitive Wyalkatchem revealed significantly more changes in both primary metabolites (25) and lipids (19) compared to cold-tolerant Young which showed only one metabolite and ten lipid changes across all the time points (Table 1). When comparing quantitative and qualitative differences in cold acclimation between the two varieties, a total of 43 metabolites and 47 lipids were found to differ across all time points (Table 1). All metabolites and lipids listed in Table 1 were further analysed with Tukey’s honestly significant difference (HSD) post hoc test in order to determine which pair-wise combinations showed significant differences for both primary metabolites and lipids (Tables S9, S10). Many pair-wise combinations were shown to differ significantly for both metabolites (387; highlighted in Table S9) and lipids (470; highlighted in Table S10) for the two wheat varieties and across all time points. But only some of these comparisons are relevant for identification of potential metabolite markers for chilling and frost tolerance phenotyping. Comparisons between TP_1_ versus TP_0_ (short cold treatment) and TP_3_ versus TP_1_ (stability over prolonged cold treatment during cold acclimation), as well as comparisons between the day and night-time samples (TP_2_ vs. TP_1_ and TP_4_ vs. TP_3_; effect of diurnal fluctuations on metabolite levels), and comparison between these time points for the two wheat varieties are essential to identify those metabolites that could be used as stable markers for cold-tolerance phenotyping. To achieve this particular aim we performed additional Student’s *t*-test to carry out pair-wise comparisons to identify candidate metabolite markers that are able to differentiate the response in cold acclimation and cold tolerance between the two wheat varieties.

**Table 1:** Metabolites and lipids that changed significantly over the 4-day of cold stress in the flag leaves of cold-sensitive Wyalkatchem (W) and cold-tolerant Young (Y), analyzed by One-way ANOVA with FDR-adjusted p < 0.05 as significance, followed by Tukey Test

Flag leaves_within variety				Flag leaves_between the two varieties	
Wyalkatchem		Young		Wyalkatchem versus Young	
Metabolites	Lipids	Metabolites	Lipids	Metabolites	Lipids
				2-cis-4-trans-Abscisic acid	LPC(30:6)
2-Oxoglutarate	PC(32:0)		PC(32:0)	2-Oxoglutarate	PC(32:0)
			PC(32:1)	Aconitate	PC(32:1)
Alanine				Alanine	PC(32:2)
				Arabinose	PC(33:1)
	PC(33:2)			Arabitol	PC(33:2)
Arginine				Arginine	PC(33:3)
Asparagine				Asparagine	PC(33:4)
beta-Alanine	PC(34:0)		PC(34:0)	beta-Alanine	PC(34:0)
Citrate	PC(34:1)		PC(34:1)	Citrate	PC(34:1)
Citrulline				Citrulline	PC(34:2)
Fructose	PC(34:3)		PC(34:3)	Fructose	PC(34:3)
				Fructose-6-P	PC(34:4)
GABA	PC(35:0)			GABA	PC(35:0)
	PC(35:1)			Galactitol	PC(35:1)
Glucose				Glucose	PC(35:2)
Glucose-6-P			PC(35:3)	Glucose-6-P	PC(35:3)
Glycine				Glycine	PC(35:4)
Histidine				Histidine	PC(35:5)
				Homoserine	PC(36:1)
	PC(36:2)			Inositol	
	PC(36:3)			Isocitrate	PC(36:3)
Isoleucine	PC(36:4)			Isoleucine	PC(36:4)
Leucine	PC(36:6)			Leucine	PC(36:6)
Lysine				Lysine	PC(37:4)
	PC(38:3)			Maltose	PC(38:3)
Mannose	PC(38:4)			Mannose	PC(38:4)
				Methionine	PC(38:6)
Phenylalanine				Phenylalanine	PC(39:3)
Proline				Proline	PC(41:6)
Putrescine				Putrescine	PE(34:3)
				Quinate	PE(36:4)
				Raffinose	PE(36:6)
				Ribose	PE(38:3)
				Salicylic acid	PE(38:6)
				Serine	PE(40:10)
Shikimate		Shikimate	PG(32:0)	Shikimate	PG(32:0)
Sucrose				Sucrose	PG(32:1)
	PG(34:1)			trans-Cinnamic acid	PG(34:1)
	PG(34:2)			Trehalose	PG(34:2)
Tryptophan	PG(34:3)				PG(34:3)
Tyrosine				Tyrosine	PG(36:4)
Valine	PG(36:5)		PG(36:5)	Valine	PG(36:5)
				Xylose	PG(36:6)
					PI(34:3)
	PI(36:4)				PI(36:4)
			PI(36:5)		PI(36:5)
	PI(36:6)		PI(36:6)		PI(36:6)

### A 4-day chilling time course reveals significant differences in cold acclimation in Wyalkatchem and Young flag leaves

#### *Sample comparisons: TP*_*1*_*versus TP*_*0*_*, TP*_*2*_* versus TP*_*1*_*, TP*_*3*_*versus TP*_*1*_* and TP*_*4*_*versus TP*_*3*_* for both varieties*

After the first night of cold exposure, the sensitive variety Wyalkatchem showed the most dramatic changes in primary metabolite levels (WTP_1_ vs. WTP_0_). As shown in Fig. 1, seven amino acids and amines were significantly decreased, including alanine (− 1.9-fold), beta-alanine (− 1.7-fold), glycine (− 2.9-fold), methionine (− 1.7-fold), phenylalanine (− 2.9-fold), proline (− 2.7-fold) and valine (− 2.3-fold). One sugar increased significantly at TP_1_: glucose-6-phosphate (+ 1.8-fold). There was no significant change in phytohormones. Three lipids increased significantly after overnight cold exposure of Wyalkatchem (Fig. 2): PC(34:0) (+ 1.6-fold), PC(34:1) (+ 1.6-fold) and PG(36:5) (+ 1.4-fold). In contrast to Wyalkatchem, the cold tolerant variety Young did not show any significant changes in primary metabolites or lipids at TP_1_ compared to TP_0_ (Figs. 1, 2).

To test for recovery and the effect of diurnal fluctuations on metabolite levels we harvested the TP_2_ samples 6 h after the TP_1_ samples, when plants were allowed to experience day-light and normal temperatures (21 °C) for 6 h. Comparison of TP_2_ and TP_1_ metabolite levels revealed that some of the significant changes we observed at TP_1_ in Wyalkatchem flag leaves were partially reversed, but some were maintained and new changes also appeared (Table S5, Fig. S2). Some amino acids and amine levels changed from being decreased at TP_1_ to being significantly increased at TP_2_ and regaining T_0_ levels: citrulline (+ 14.9-fold), glycine (+ 2.3-fold), homoserine (+ 2.2-fold), methionine (+ 1.6-fold) and putrescine (+ 3.0-fold). Tryptophan decreased further at TP_2_ compared to TP_1_: (− 1.9-fold). There were no differences in phytohormone levels, nor were there any significant changes in lipid levels between TP_2_ and TP_1_ Wyalkatchem flag leaves (Table S6, Fig. S3). Comparing TP_2_ to TP_1_ in Young showed a significant increase for citrulline (+ 2.8-fold) (Table S5, Fig. S2). There were no significant lipid changes (Table S6, Fig. S3).

After prolonged four-night cold treatment (TP_3_), more significant accumulation of sugars and organic acids were observed in Wyalkatchem flag leaves compared to the first overnight exposure to cold (WTP_1_; Fig. 1). Fructose (+ 4.1-fold) and glucose (+ 6.0-fold) increased significantly in Wyalkatchem flag leaves. Also, arabinose (+ 1.3-fold) and mannose (+ 1.4-fold) were higher compared to TP_1_. Two organic acids, quinate (+ 1.5-fold) and shikimate (+ 2.3-fold), and one amine, putrescine (+ 1.8-fold), were significantly higher. One amino acid, asparagine (− 3.9-fold) was markedly lower at WTP_3_ compared to WTP_1_. Prolonged cold treatment of Wyalkatchem also resulted in more lipid changes at WTP_3_ compared to WTP_1_. Four lipids were significantly reduced, one being a saturated species [PC(32:0)] and the remaining three being unsaturated species [PC(32:1), PC(36:6), PI(36:6)]. Another 13 lipids increased significantly; one of them was a saturated lipid, while 12 others were unsaturated species (Fig. 2, Table S6). In Young, none of the primary metabolites were significantly changed at YTP_3_ compared to YTP_1_ (Fig. 1). The most notable changes after prolonged cold stress (YTP_3_) in Young flag leaves compared to the shorter cold stress (YTP_1_) were in the lipid levels. Fifteen lipids were significantly reduced compared to TP_1_, while one polyunsaturated lipid species, PC(38:2), was significantly higher (1.4-fold). Of the 15 lipids with reduced levels, two were saturated species while a majority (13) were unsaturated species (Fig. 2, Table S6). Comparing TP_4_ day-time samples to TP_3_ in Wyalkatchem and Young revealed that there were no significant differences in primary metabolites (Table S5, Fig. S2), while only one polyunsaturated lipid species, PC(41:6) was increased (+ 1.5-fold) in Young flag leaves (Table S6, Fig. S3).

### Young and Wyalkatchem show significant varietal differences in cold acclimation across time points

#### *Sample comparisons: WTP versus YTP at each time point*

**Fig. 5:**
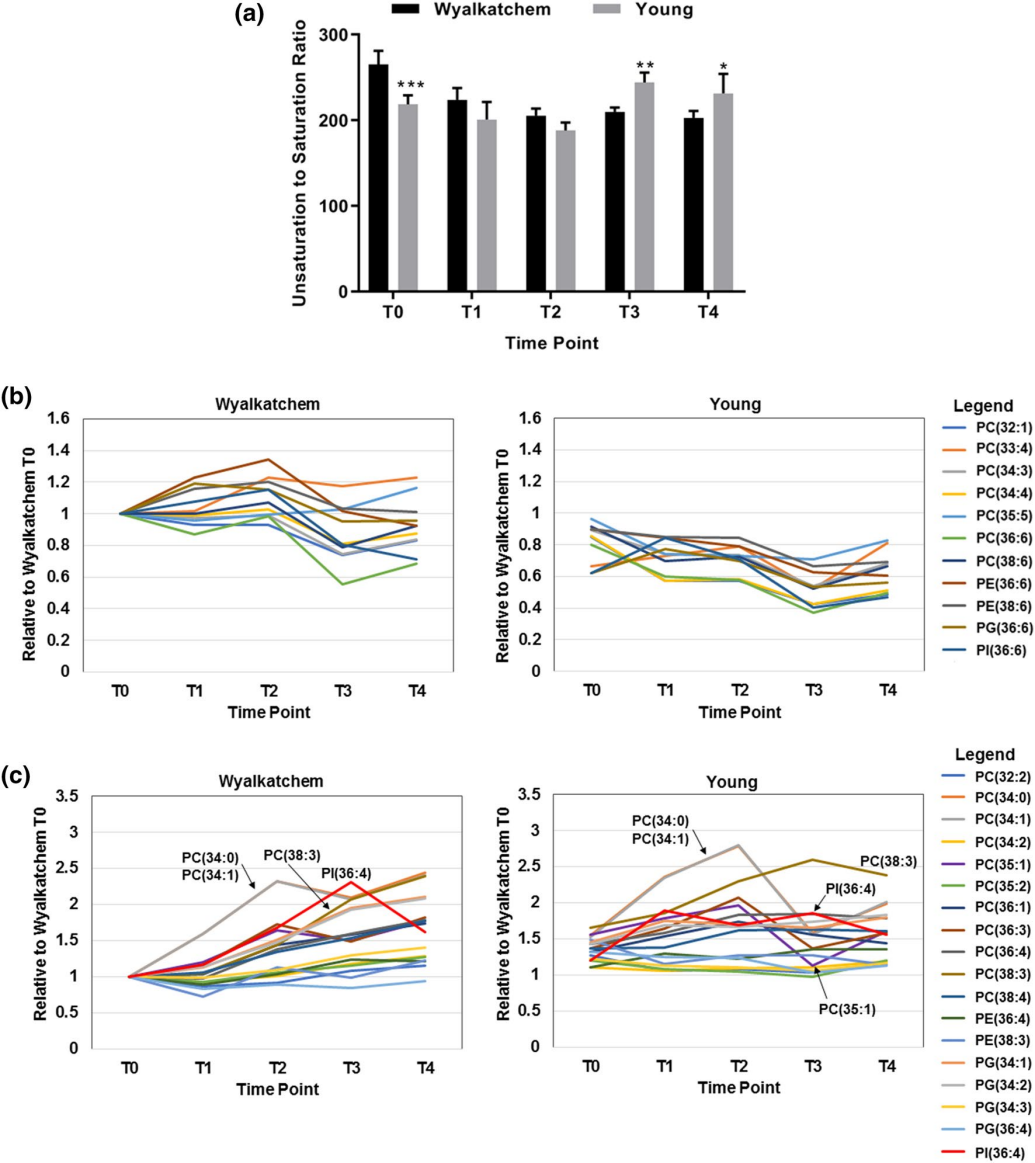
**a** Unsaturation to saturation ratio in Wyalkatchem and Young across the time points. Student’s t-test was used to compare the ratio values between Wyalkatchem and Young at each time point. Error bars indicate the standard deviation (SD) of four biological replicates (n = 4) and asterisks indicate significance levels: *p < 0.05, **p < 0.01 and ***p < 0.001. **b** Expression levels of a group of 11 lipids with higher expression from TP_1_ onwards in the cold-sensitive Wyalkatchem compared to Young [(PC(32:1), PC(33:4), PC(34:3), PC(34:4), PC(35:5), PC(36:6), PC(38:6), PE (36:6), PE(38:6), PG(36:6), PI(36:6)]. These lipids had higher levels in Wyalkatchem after the first cold treatment and most of them remained higher than in Young throughout the treatment. **c** Expression levels of a group of 18 lipids with higher expression from TP_1_ onwards in the cold-tolerant Young compared to Wyalkatchem [(PC(32:2), PC(34:0), PC(34:1), PC(34:2), PC (35:1), PC(35:2), PC(36:1), PC(36:3), PC(36:4), PC(38:3), PC(38:4), PE(36:4), PE(38:3), PG(34:1), PG(34:2), PG(34:3), PG(36:4), PI(36:4)]. Arrows indicate three lipids, PC(34:0), PC(34:1) and PC(35:1), that changed drastically from TP_2_ onwards in Young. **b** and **c** illustrate how these lipids behaved after long term exposure to cold and how they respond to diurnal rhythms

In the unstressed plants (TP_0_), there were no significant differences in the levels of primary metabolites or lipids between Wyalkatchem and Young flag leaves (Figs. 3, 4). However, several metabolites with potential roles in stress responses were slightly higher in Young compared to Wyalkatchem: sucrose (+ 1.4-fold), mannitol (+ 1.8-fold), GABA (+ 3.1-fold), ABA (+ 1.7-fold). At TP_0_, there were no significant differences between Wyalkatchem and Young in terms of lipid content. However, some lipid levels appeared to be different in Wyalkatchem and Young flag leaves. Some lipids were lower in Young compared to Wyalkatchem when a cut-off of 0.1 was used: PC(33:4) (− 1.5-fold), PG(36:6) (− 1.6-fold) and PI(36:6) (− 1.6-fold). Others were higher in Young compared to Wyalkatchem (*p *< 0.1): PC(35:1) (+ 1.5-fold), PG(34:1) (+ 1.4-fold) and PG(34:2) (+ 1.4-fold) (Fig. 4). Although these lipids (indicated by asterisks) did not satisfy the significance threshold (*p *< 0.05) at TP_0_, they did become significantly different (*p *< 0.05) after the first night of cold treatment (TP_1_; Fig. 4). This may indicate that the lipid composition in Wyalkatchem and Young may be different before cold treatment. This is also reflected by the fact that at TP_0_, the unsaturated to saturated lipid ratio of Young (218.6 ± 10.6) was significantly lower than in Wyalkatchem flag leave samples (265.2 ± 16.0; Fig. 5a, Table S11).

After overnight exposure to cold (TP_1_), two amino acids and amines were significantly higher in Young compared to Wyalkatchem (Fig. 3): homoserine (+ 2.2-fold) and methionine (+ 2.4-fold). Trehalose (+ 2.2-fold) was the only sugar that was significantly higher in Wyalkatchem flag leaves. The phytohormone salicylic acid decreased in Young compared to Wyalkatchem (− 2.4-fold). Lipids showed the most dramatic difference between the two varieties at TP_1_. Fourteen lipid species were significantly higher in Young TP_1_ samples and all were polyunsaturated lipids. Thirteen lipid species were significantly higher in Wyalkatchem compared to Young, and all were also unsaturated lipids (Fig. 4, Table S6). The lipid unsaturation to saturation ratio was not significantly different between Wyalkatchem and Young flag leaves at this time point (Fig. 5a). When comparing the TP_2_ samples between the two wheat varieties (YTP_2_ vs. WTP_2_), two metabolites in Young were significantly lower compared to Wyalkatchem (Fig. 3): trehalose (− 3.0-fold), and asparagine (− 2.4-fold). None of the lipid species were significantly different in Young compared to Wyalkatchem at this time point (Fig. 4, Table S6).There was no significant difference in the lipid unsaturation to saturation ratio between the two varieties at TP_2_ (Fig. 5a, Table S11).

After prolonged cold stress exposure (TP_3_; Fig. 3), some metabolites were significantly lower in Young compared to Wyalkatchem, including arabinose (− 1.4-fold), fructose (− 4.2-fold), glucose (− 5.7-fold), mannose (− 1.5-fold), quinate (− 1.6-fold), shikimate (− 2.4-fold), putrescine (− 2.4-fold) and salicylic acid (− 2.1-fold). Comparison of the lipids at TP_3_ again showed the biggest difference between the two varieties. Twenty-five lipid species were significantly lower at TP_3_ in Young compared to Wyalkatchem (Fig. 4, Table S6). The total lipid unsaturation to saturation ratio was significantly higher in Young compared to Wyalkatchem at this stage (Fig. 5a, Table S11). Comparison of TP_4_ samples between Young and Wyalkatchem (YTP_4_ vs. WTP_4_) showed that only three metabolites were significantly lower in Young compared to Wyalkatchem (Fig. 3): fructose (− 2.6-fold), quinate (− 1.8-fold) and shikimate (− 3.0-fold). In addition, six lipids were significantly lower in Young when compared to Wyalkatchem (Fig. 4, Table S6). They were PC(32:1), PC(34:4), PC(35:4), PC(38:6), PG(32:0) and PG(36:6). All of these six lipids were the same as those found to be lower in Young at previous time points (TP_1_ to TP_3_). The unsaturation to saturation ratio of Young was again significantly higher than Wyalkatchem at this stage (Fig. 5a, Table S11).

### Quantitative changes in two groups of lipids may account for differences in membrane fluidity and cold tolerance between Young and Wyalkatchem

The time course experiment revealed that the unsaturated to saturated lipid ratio changed progressively from being higher in Wyalkatchem compared to Young at TP_0_, to higher in Young at TP_3_ and TP_4_ (Fig. 5a). This shift in unsaturated to saturated lipid ratio is correlated with differences in the expression profile of two main groups of lipids from TP_0_ onwards (Fig. 5b, c). The first group of 11 lipids was higher in Wyalkatchem after the first cold exposure (TP_1_) and remained higher than in Young throughout the 4-day time course (Fig. 5b, Table S12). In contrast, another group of 18 lipids (Fig. 5c, Table S13) were on average higher in Young compared to Wyalkatchem from the first exposure to cold onwards (TP_1_), but 5 members of this group behaved quite differently in the two wheat varieties (Fig. 5c). The saturated species PC(34:0), monounsaturated species PC(34:1) and PC (35:1) were significantly increased in both Wyalkatchem and Young after one overnight cold stress event (TP_1_), but were then reduced drastically in Young compared to Wyalkatchem after the fourth night of cold stress (TP_3_). A fourth lipid, PI(36:4), was increased in Young from time points TP_1_ to TP_3_, but was induced more strongly in Wyalkatchem after prolonged cold exposure (TP_3_). The unsaturated lipid species PC(38:3) was always significantly higher in Young compared to Wyalkatchem from TP_0_ onwards and is gradually increased during prolonged cold exposure and was not subjected to diurnal fluctuations (TP_2_ and TP_4_). PC(38:3) was only increased in Wyalkatchem from TP_3_ onwards. The combined changes in these two lipid groups and the differential expression behaviour of some members in these lipid groups may contribute towards the observed increase in the ratio of unsaturated to saturated lipids in Young compared to Wyalkatchem (Fig. 5a, Table S11).

## Discussion

### Wheat chilling and frost tolerance: field versus controlled environment phenotyping

In the field, phenotyping for wheat frost tolerance is complicated by the unpredictability and spatial/temporal variation in the severity of frost events (Frederiks et al. 2012). Field phenotyping has focused on the presence/absence of tissue damage and yield effects caused by frosts. Frost events at the critical stage of flowering are often catastrophic, leading to the assumption that there is little genetic variation for frost tolerance in wheat (Frederiks et al. 2012; Zheng et al. 2015; Barlow et al. 2015). Non-freezing, chilling conditions occur far more frequently in the field, but the associated sterility and loss in spike grain number has received less attention (Subedi et al. 1998a, b; Chakrabarti et al. 2011; Smith and Zhao 2016). In rice, cold-induced pollen sterility causing loss in grain yield is commonly used as a phenotyping trait (Hayase et al. 1969; Satake et al. 1969; Nishiyama 1984; Oliver et al. 2005). Similar to drought and heat stress, phenotyping for chilling and frost tolerance in the field is compromised by avoidance or escape mechanisms such as phenology of flowering (Fleury et al. 2010; Richards et al. 2010; Shavrukov et al. 2017). Controlled environment phenotyping can overcome some of the pitfalls associated with field work. Occurrence and severity of cold events can be monitored and repeated consistently, as well as their timing during reproductive growth controlled, making it possible to study the physiological and molecular basis of cold tolerance without the interference of avoidance or escape mechanisms or the complexity of changes in frost severity and duration between events. We established a controlled environment screening method based on maintenance of grain number under chilling conditions using two wheat cultivars that were reproducibly shown to be more tolerant (Young) and sensitive (Wyalkatchem) to field chilling and frost conditions. This screening method allowed us to reproduce the field rankings for the two cultivars, as well as the cold tolerance rankings for several other tolerant and sensitive wheat cultivars from the National Frost Initiative (Dolferus et al., unpublished results). Chilling conditions led to a reduction in spike grain number in wheat and we show that the YM and anthesis stages are the two most sensitive stages to chilling stress.

### Metabolomics as alternative phenotyping tool for chilling and frost tolerance in wheat

Tolerance to frost requires the establishment of an acclimation response in plant tissues. Cold acclimation starts below a certain non-freezing threshold temperature before below-zero temperatures cause ice formation and desiccation and freezing damage to plant tissues (Livingston et al. 2016). It remains unclear how much time it takes for effective cold acclimation to reach maximal potential, nor do we know whether there is genetic variation in quantitative or qualitative aspects of cold acclimation (Pagter and Arora 2013; Vanková et al. 2014; Chen et al. 2014; Fiebelkorn and Rahman 2016). Understanding what acclimation to non-freezing or chilling conditions involves is essential to understand how frost tolerance (cold, chilling/dessication and freezing) can be improved.

Acclimation to abiotic stresses such as cold leads to accumulation of metabolites in plant tissues to protect cellular functions (Morgan 1992; Ruelland et al. 2009; Ouellet and Charron 2013; Miura and Furumoto 2013). Some of these compounds act as osmoprotectants to protect against abiotic stresses that affect the water balance of plants (Vágújfalvi et al. 1999; Uemura et al. 2003; Chen et al. 2014). Osmolytes are small, electrically neutral, water-soluble organic compounds that efficiently maintain osmotic balance and stabilize membranes and macromolecules under water stress conditions. They include betaines, amino acids, polyols and non-reducing sugars (Burg and Ferraris 2008; Slama et al. 2015; Nahar et al. 2016; Argiolas et al. 2016). Osmolytes also protect membranes and act as scavengers for toxic reactive oxygen species. They are induced by a variety of other abiotic stresses that affect the water balance (drought, heat, salinity), making them therefore less specific markers for cold tolerance per se (Beck et al. 2007). There is no clear evidence available as to how quantitative or qualitative differences in accumulation of these compounds directly contribute to cold tolerance. We compared changes in the concentrations of metabolites and lipids during cold acclimation for two wheat varieties that differ in cold tolerance. Many metabolites are subject to day-night circadian fluctuations and cold or frost events usually occur during the night. It has been demonstrated that there is a substantial overlap between cold and circadian-regulated genes, suggesting that cold acclimation is tightly linked to circadian rhythms (Espinoza et al. 2010; Sanchez et al. 2011). However, it is expected that some compounds that accumulate after an initial chilling or frost event during the night remain present in plant tissues as a protection against subsequent chilling events. To investigate this, we designed an experiment where we compared metabolite levels in leaves harvested in the morning immediately after a chilling event and 6 h later, when plants were allowed to recover in the light at normal temperatures. We also compared these samples for a single and four consecutive chilling cycles. Because our focus is on using metabolites as diagnostic markers for cold acclimation, we first used the easy-to-collect flag leaves.

### Effect of prolonged/consecutive chilling treatments on wheat flag leaf metabolites and lipids

Cumulative changes with respect to TP_0_, TP_1_ and TP_3_ for both Wyalkatchem and Young flag leaves show significant changes occurring after the first cold night (TP_1_), with additional changes appearing after prolonged cold treatment (TP_3_). However, fewer changes were observed in the day-time samples (TP_2_ and TP_4_), suggesting that partial recovery takes place during normal day temperatures for some metabolites. Day-night fluctuation in levels of some metabolite is likely due to circadian rhythms and fluctuation of photosynthetic activity and associated metabolism and needs to be taken into account for field sampling and cold tolerance phenotyping. After the first night of cold treatment, a significant reduction in the levels of most amino acids and amines was evident in Wyalkatchem, but not in Young. Most amino acids and amines were higher in Young compared to Wyalkatchem from the first overnight cold treatment (TP_1_). Interestingly, the amino acids proline and citrulline, which are known to accumulate in plant tissues under a variety of stresses that affect water relations (drought, salinity, cold, heat; Mayer et al. 1990; Ashraf and Foolad 2007; Lehmann et al. 2010; Hayat et al. 2012; Liang et al. 2013), behaved quite differently in Wyalkatchem compared to Young. Proline was found to decrease significantly after one night cold treatment in Wyalkatchem but increased in Young flag leaves. Citrulline behaved in a similar fashion, but was only found to change significantly at a more relaxed cut-off of (*p *< 0.1) in both wheat lines. Citrulline has hydroxyl radical scavenging and antioxidant properties and can protect DNA and enzymes from oxidative injuries (Kawasaki et al. 2000; Akashi et al. 2001; Rimando and Perkins-Veazie 2005; Kusvuran et al. 2013).

In Young, a significant increase in citrulline was observed in the TP_2_ samples. GABA, an amine with cryo-protective properties (Bouche and Fromm 2004; Mazzucotelli et al. 2006) was also marginally increased-albeit below significance levels. After the prolonged cold treatment (TP_3_), amino acid levels in Wyalkatchem did not fully recover and citrulline increased further. Accumulation of citrulline occurred predominantly in Wyalkatchem and only temporarily in Young (TP_1_). Wyalkatchem also showed a threefold increase for the polyamine putrescine, an amino acid breakdown product with growth-regulatory properties known to play a role in abiotic stresses (Gill and Tuteja 2010; Shi and Chan 2014). Shikimate, a substrate for the synthesis of lignin and various aromatic compounds such as quinate (Herrmann and Weaver 1999; Guo et al. 2014) was increased in Wyalkatchem but decreased in Young. The enzymes of this pathway have been shown to be involved in defence responses (Kasai et al. 2005). Furthermore, compatible osmolytes such as sugars (arabinose, glucose, glucose-6-P,fructose and raffinose) and the sugar alcohol mannitol also accumulated in Wyalkatchem upon cold treatment, but not in Young flag leaves. Sugars and sugar alcohols play a role as osmo-and cryo-protectants (Loescher et al. 1992; Travert et al. 1997; Burg and Ferraris 2008; Slama et al. 2015). The accumulation of these osmolytes indicates that Wyalkatchem is responding to water or desiccation stress as a consequence of cold treatment. Repression of amino acid synthesis in Wyalkatchem may also contribute to this situation. None of these symptoms was observed in tolerant variety Young. Osmolytes can therefore serve as markers for chilling sensitivity; they may be indicative of water stress as a result of membrane damage caused by chilling and frost. But osmolytes point to a secondary effect of cold treatment and their accumulation is therefore not specific for low temperature stress. Osmolyte accumulation can be activated by other abiotic stresses as well (drought, heat, salinity; Beck et al. 2007). We did not observe many significant differences in phytohormone levels between Wyalkatchem and Young. Interestingly, the only phytohormone that showed significant differences between the two wheat varieties was salicylic acid. Trans-cinnamic acid (significant at TP_2_), one of the precursors of salicylic acid biosynthesis (Hayat et al. 2007), follows the same abundance profile as salicylic acid. Salicylic acid levels were consistently higher in Wyalkatchem compared to Young at both short and prolonged cold stress exposures. Salicylic acid is known for its role in plant pathogen responses, but also plays a role in abiotic stress in regulating stomatal closure under water stress. When applied to plants, salicylic acid was shown to reduce freezing tolerance and at high levels causes oxidative stress and increased stress sensitivity (Lissarre et al. 2010; Miura and Tada 2014; Eremina et al. 2016).

Cold treatment of Wyalkatchem and Young flag leaves resulted in quantitative and qualitative changes in the lipid composition and the lipid changes outnumber those observed for the primary metabolites. The most prevalent changes in cold-stressed Wyalkatchem and Young flag leaves were observed for the phospatidylcholine (PC) class of lipids. PCs are the most abundant plasma membrane lipids in plants and animals. Differences between Wyalkatchem and Young PC levels in response to cold may therefore contribute to changes in plasma membrane fluidity and permeability (Upchurch 2008; van Meer et al. 2008). The saturated lipid species PC(34:0), monounsaturated species PC(34:1) and PC(35:1) increased in both wheat varieties after the first exposure to cold (TP_1_) and then decreased after prolonged cold treatment (TP_3_) (Fig. 5c). However, the decrease of these PCs at TP_3_ was faster in Young compared to Wyalkatchem and this may have contributed to a higher level of unsaturated lipids in Young compared to Wyalkatchem (Fig. 5c). Even at TP_0_, Young and Wyalketchem flag leaves show a different lipid saturation:unsaturation ratio and some lipid levels are marginally different, but become significantly different after the first night of cold treatment. This seems to indicate that Young is better adapted to cold from the beginning. The change in the unsaturated to saturated lipid ratio is the result of a change in relatively few lipid species and this may be the result of a modification in the expression of a small number of genes. The fact that Young and Wyalkatchem show a different lipid content before and after cold treatment may indicate that both lines are adapted differently to cold temperatures and this may be due to genetic variation in the regulation or function of lipid biosynthesis gene/genes. Using lipids as phenotyping markers may enable us to identify those genes using a mapping population. The superior ability of the tolerant variety Young to adjust and remodel its membrane composition may be the key factor to overcome prolonged cold exposure. We do not know to what extend the differences in lipid content between Young and Wyalkatchem flag leaves can protect against frost (temperature threshold) or whether the observed differences are sufficient to survive more severe frost conditions. But using lipidomics to screen wheat germplasm collections for varieties with higher lipid unsaturation levels than Young may hold the key to improving frost tolerance in wheat. Plants produce a large variety of lipid species in their chloroplasts and the fact that the spectrum of synthesised lipids depends significantly on the reigning environmental conditions (e.g., temperature) indicates that modification of membrane lipid composition is an important environmental adaption mechanism (Aid 2019).

Phosphatidylglycerol lipids (PGs) are the main phospholipid class of thylakoid membranes in higher plants (Ren et al. 2014). PGs are the second largest lipid group that is affected by cold in wheat flag leaves. The saturated lipid PG(32:0) is lower in Young and higher in Wyalkatchem flag leaves after the prolonged cold stress (TP_3_), indicating that this lipid species may also contribute to the higher degree of lipid unsaturation in membranes of the tolerant variety Young. Phosphotidylinositol (PI) lipids are the third most abundant extraplastidic lipid. PIs are involved in membrane signalling under abiotic stress conditions such as osmotic stress (Munnik and Vermeer 2010; Zheng et al. 2016). PIs increase by 50% after freezing in Arabidopsis and contribute to frost tolerance (Zheng et al. 2016). Significantly higher levels in Young compared to Wyalkatchem of polyunsaturated PI species such PI(36:4) at TP_1_ may also contribute to better membrane protection for the tolerant variety during cold stress.

Plants respond with complex changes in lipid composition during cold acclimation to maintain membrane integrity (Uemura and Steponkus 1994; Uemura et al. 1995; Degenkolbe et al. 2012). The amount of double bonds in unsaturated lipids improves membrane fluidity at lower temperatures. To maintain optimum membrane fluidity, unsaturation levels of lipids decreases at higher temperatures and increases at lower temperatures (Narayanan et al. 2016a, b; Zheng et al. 2016). The significant increase in the ratio of unsaturated to saturated lipids after prolonged cold treatment suggests that Young is better able to adapt its membrane composition to low temperatures. Failure of Wyalkatchem to modify lipid composition in favour of unsaturated to saturated lipids may lead to membrane damage, causing electrolyte leakage and water loss, leading to the osmotic stress response observed in this variety. The lipid changes we observed in Young and Wyalkatchem flag leaves are part of an acclimation response to cold; they are amplified during prolonged cold treatment, they are not subjected to diurnal fluctuations and they are specific for low temperature adaptation. Lipids are therefore suitable markers for chilling and frost tolerance phenotyping.

## Conclusion

In conclusion, combined metabolome and lipidome analyses in flag leaves have shed light on important differences in cold acclimation between the cold-sensitive Wyalkatchem and cold-tolerant Young varieties. The main differences between the two wheat cultivars were the differential modification of membrane lipids in both varieties and the establishment of an osmotic stress-like response in the sensitive varieties Wyalkatchem. There is a gradual change in the ratio of unsaturated versus saturated lipids that favors higher levels of unsaturated lipids being incorporated in the tolerant variety Young. As a consequence, Young may acquire a superior ability to adapt membrane fluidity to cold conditions and avoid membrane damage. Diagnostic metabolites, including sugars and lipids, can therefore serve as tools to improve the reliability of phenotyping for chilling and frost tolerance in wheat. Furthermore, metabolomics and lipidomics can be combined with transcriptome analysis and genomics approaches to identify the genetic mechanism driving the observed differences in metabolite accumulation and this could lead to future DNA marker-based approaches for frost tolerance breeding in wheat.

## Electronic supplementary material

Below is the link to the electronic supplementary material.

Supplementary material 1 (DOCX 103 kb)

Supplementary material 2 (DOCX 2792 kb)

Supplementary material 3 (XLSX 837 kb)

## Data Availability

All the data sets that support the findings of this study are available as Supplemental data files (Microsoft Excel 2013 files).
