# Supplementary material for: Phenotyping reproductive stage chilling and frost tolerance in wheat using targeted metabolome and lipidome profiling
Source: Metabolomics. 2019 Oct 20;15(11):144. doi: 10.1007/s11306-019-1606-2 (PMC6800866; doi:10.1007/s11306-019-1606-2)

**Supplementary Data S1: Methodology for Phytohormone Analysis**

1. Preparation of calibration and internal standards for the ten phytohormones and organic acids [Three organic acids that serve as precursors for phytohormones: benzoic acid (BA), *trans*-Cinnamic acid (CA), 12-oxo phytodienoic acid (OPDA); Seven phytohormones: Salicylic acid (SA), Jasmonic acid (JA), Jasmonoyl-isoleucine (JA-ile), Indole-3-acetic acid (IAA), Indole-3-carboxylic acid (ICA), Indole-3-butyric acid (IBA), 2-cis-4-trans-Abscisic acid (ABA)]
2. Preparation of phytohormone calibration standard mixture, 5 ug/ml

M1 = 50 ug/ml original concentration for each phytohormone and organic acid,

V1 = ? ul, M2 = 5, V2 = 2000 uL

M1V1 = M2V2

- V1 = 200 uL
- Take each 200 uL of the 10 phytohormones and organic acid to make the 2000 uL mixture, kept at -80°C

1. Preparation of phytohormone calibration standard mixture, 0.5 ug/ml

M1 = 5 ug/ml, V1 = ?, M2 = 0.5 ug/ml, V2 = 1000 uL

M1V1 = M2V2

- V1 = 100 uL
- Take 100 uL of **A** and 900 uL MEOH (methanol) to make the 1000 uL mixture, kept at -80°C

1. Preparation of phytohormone calibration standard 0.05 ug/ml

M1 = 0.5 ug/ml, V1 = ?, M2 = 0.05 ug/ml, V2 = 1000 uL

M1V1 = M2V2

- V1 = 100 uL
- Take 100 uL of **B** and 900 uL MEOH to make the 1000 uL mixture, kept at -80°C

1. Preparation of phytohormone calibration standard 0.005 ug/ml

M1 = 0.05 ug/ml, V1 = ?, M2 = 0.005 ug/ml, V2 = 1000 uL

M1V1 = M2V2

- V1 = 100 uL
- Take 100 uL of **B** and 900 uL MEOH to make the 1000 uL mixture, kept at -80°C

1. Preparation of ISTD (internal standard) mixture (d_2_IAA, d_5_BA, d_6_ABA, d_6_SA, d_7_CA, H_2_JA)

M1 = 50 ug/ml, V1 = 100 uL, M2 = ?, V2 = 600 uL

M1V1 = M2V2

- M2 = 8.3 ug/ml
- Take each 100 uL of the 6 ISTD to make the 600 uL mixtures, kept at -80°C
- (the final ISTD conc. in each calibration standard is 145.3 ng/ml, by adding 7 ul of 8.3 ug/ml stock mixtures in a total volume of 400 ul, refer to the table below)

**F**) Valine ISTD, kept in -80°C until use

M1 = 50 ug/ml, V1 = 100 uL, M2 = ?, V2 = 600 uL

M1V1 = M2V2

- M2 = 8.3 ug/ml
- Take each 100 uL of the M1 and top up with 500 ul MEOH to make the 600 uL mixtures, kept at -80°C
- (the final Valine ISTD conc. in each calibration standard is 145.3 ng/ml, by adding 7 ul of 8.3 ug/ml stock mixtures in a total volume of 400 ul, refer to the table below)

1. Preparation of phytohormone and organic acids calibration standards (containing ISTD)

| Calibration standard (ug/ml) | **A)** 5 ug/ml stock mixture (uL) | **B)** 0.5 ug/ml stock mixture (uL) | **C)** 0.05 ug/ml stock mixture (uL) | **D)** 0.005 ug/ml stock mixture (uL) | **E)** ISTD stock mixture 8.3 ug/ml (uL) | **F)** Valine ISTD stock 8.3 ug/ml (uL) | 1 % NaOH (uL)  +  suspend | MEOH(uL) | Pyridine (uL)  +  Vortex vigorously for 25-30s |
| --- | --- | --- | --- | --- | --- | --- | --- | --- | --- |
| 0.00025 |  |  |  | 20 | 7 | 7 | 200 | 133 | 34 |
| 0.0005 |  |  |  | 40 | 7 | 7 | 200 | 113 | 34 |
| 0.001 |  |  |  | 80 | 7 | 7 | 200 | 73 | 34 |
| 0.0025 |  |  | 20 |  | 7 | 7 | 200 | 133 | 34 |
| 0.005 |  |  | 40 |  | 7 | 7 | 200 | 113 | 34 |
| 0.01 |  |  | 80 |  | 7 | 7 | 200 | 73 | 34 |
| 0.025 |  | 20 |  |  | 7 | 7 | 200 | 133 | 34 |
| 0.05 |  | 40 |  |  | 7 | 7 | 200 | 113 | 34 |
| 0.1 |  | 80 |  |  | 7 | 7 | 200 | 73 | 34 |
| 0.25 | 20 |  |  |  | 7 | 7 | 200 | 133 | 34 |
| 0.5 | 40 |  |  |  | 7 | 7 | 200 | 113 | 34 |
| 1 | 80 |  |  |  | 7 | 7 | 200 | 73 | 34 |
| 2 | 153 |  |  |  | 7 | 7 | 200 | 0 | 34 |

1. Extraction of the phytohormones and organic acids from the flag leaf tissues of Wyalkatchem and Young

The extraction of phytohormones from the leaf samples was carried out according to Cao et al., (2017) with some modifications. Briefly, the samples were ground with pestle and mortar in liquid nitrogen and frozen immediately. Then, a 100 mg was weighed for each of the sample and put into a 2 ml centrifuge tube. 1 ml of extraction solvent (containing 956 µL of 70% methanol and 44 µL of 8.3 ug/ml ISTD mixture) was added to the sample, vortexed and extracted with a thermoshaker at 4 °C, 1400 rpm for 30 minutes. The samples were centrifuged at 16,100 X g for 10 mins. The supernatants were taken and transferred to new centrifuge tubes. The residues were extracted again with 500 µL of extraction solvent (without ISTD) with the same extraction steps. The first and second supernatants were combined and kept at – 80 °C until further use.

1. Derivatization of samples

For the methyl-chloroformate derivatization of the samples, the protocols were referred to Rawlinson et al., (2015) with some modifications. Briefly, 600 µL of the supernatant for each sample was taken, dried down and re-dissolved with 160 µL methanol. Then, 8.3 ug/ml of ^13^C5, N-labelled L-Valine (another internal standard) was added. 34 µL of pyridine was added to the mixture and vortexed vigorously for 25-30s. After that, 200 µL of 1% NaOH solution was added to the mixture and suspended. This 400 µL mixture was derivatized with 20 µL of methyl chloroformate (MCF) and vortexed vigorously for 25-30s. Another 20 µL of methyl chloroformate (MCF) was added and repeated the vortex. Next, 400 µL of chloroform was added to the mixture and vortexed for 10s, followed by an addition of 400 µL of 50 mM sodium bicarbonate. The mixture was vortexed for 10-15s and centrifuged for 30s at 16,100 xg. The upper aqueous layer was discarded and the bottom organic layer was kept at -80 °C until further analysis.

1. Quantification and analysis of samples using GC-Triple Quadruple-MS

For the quantification of phytohormones, samples (1 µL) were injected into a GC–QqQ–MS system comprising of a Gerstel 2.5.2 Autosampler, a 7890A Agilent gas chromatograph and a 7010 Agilent triple-quadrupole MS (Agilent, Santa Clara, USA) with an electron impact (EI) ion source. The GC was operated in constant pressure mode (20 psi) with helium as the carrier gas. The MS was adjusted according to the manufacturer’s recommendations using tris-(perfluorobutyl)- amine (CF43). A J&W Scientific VF-5MS column (30m long with 10m guard column, 0.25mm inner diameter, 0.25um film thickness) was used. The injection temperature was set at 250 °C, the MS transfer line at 250 °C, the ion source was adjusted to 250 °C and the quadrupole at 150 °C. Helium was used as the carrier gas at a flow rate of 1mLmin^−1^. Nitrogen (UHP 5.0) was used as the collision cell gas at a flow rate of 1.5mLmin^−1^. Helium (UHP 5.0) was used as the quenching gas at a flow rate of 2.25mLmin^−1^. The following temperature program (Rawlinson et al., 2015) with modification was used; injection at 40 ◦C, hold for 1 min, followed by a 20 °Cmin^−1^ oven temperature and ramped to 255 ◦C, followed by a 15 °Cmin^−1^ oven temperature and ramped to 282 ◦C, then back to 20 °Cmin^−1^ oven temperature and ramped to 320 ◦C and a final hold for 2 min. The total run time was 17.583 min. For method optimization, calibration and internal standards were subsequently analyzed on the GC–QqQ–MS to obtain the retention times and to identify a corresponding unique precursor ion. For each precursor ion, two product ion scans were carried out using four collision energies (0, 5, 10 and 20 V) to identify product ions in which two product ions were identified. Subsequently, for the two generated products the collision energies for each major reaction monitoring (MRM) transition was optimized using a series of collision energies (CEs) between 0 and 30 V. Collision energy optimization plots for each compound are presented in **Point 6** below. Once collision energies were optimized for each MRM transition, a product ion was selected as the corresponding target ion (T) and the subsequent MRM transition was deemed as the qualifier ion (Q). Absolute concentrations (ug/ml) of targeted phytohormones/organic acids were quantified using a MRM target ion based on the linear response of the calibration. Together, all the ten phytohormones and organic acids were detected from the flag leaf samples (refer to the chromatogram in **Point 7** below). However, only six of them [ trans-Cinnamic acid (CA), Salicylic acid (SA), Jasmonic acid (JA), Jasmonoyl-isoleucine (JA-ile), Indole-3-acetic acid (IAA) and 2-cis-4-trans-Abscisic acid (ABA)] were managed to be quantified in this study. Benzoic acid (BA) although was detected in high amount, but this was largely due to the contribution from the LC-MS grade methanol solvent. Meanwhile, and 12-oxo phytodienoic acid (OPDA), Indole-3-carboxylic acid (ICA) and Indole-3-butyric acid (IBA) were absent or present in low amount in some of the samples, thus couldn’t be quantified.

1. Multiple Reaction Monitoring (MRM) developed for the phytohormone and organic acid analyses

|  | Compound name | Internal standard (/) | Precursor Ion (m/z) | Product ion | Collision Energy | Retention Time (min) |
| --- | --- | --- | --- | --- | --- | --- |
| **Quantifier** | Benzoic acid (BA) |  | 105 | 77.1 | 16 | 6.450 |
| Qualifier 1 |  |  | 105 | 51 | 28 |  |
| Qualifier 2 |  |  | 136.1 | 105 | 8 |  |
| Qualifier1 | D_5_BA | / | 110.1 | 54.1 | 28 | 6.432 |
| **Quantifier** |  | / | 110.1 | 82.1 | 16 |  |
| **Quantifier** | Valine-^13^C_5_,^15^N | / | 134.9 | 103.1 | 12 | 7.58 |
| Qualifier1 |  | / | 134.9 | 75.1 | 14 |  |
| Qualifier2 |  | / | 117.9 | 86 | 5 |  |
| Qualifier3 |  | / | 103.1 | 59.1 | 6 |  |
| **Quantifier** | trans-Cinnamic acid (CA) |  | 131.1 | 103.1 | 12 | 8.604 |
| Qualifier1 |  |  | 162.1 | 103.1 | 28 |  |
| Qualifier2 |  |  | 162.1 | 131 | 28 |  |
| **Quantifier** | D_7_CA | / | 138.1 | 82.1 | 28 | 8.582 |
| Qualifier2 |  |  | 169.1 | 110.1 | 28 |  |
| Qualifier1 |  |  | 138.1 | 110.1 | 16 |  |
| **Quantifier** | Salicylic acid (SA) |  | 135.1 | 77.1 | 16 | 9.337 |
| Qualifier1 |  |  | 135.1 | 92 | 20 |  |
| Qualifier2 |  |  | 121 | 93 | 8 |  |
| Qualifier3 |  |  | 121 | 65.1 | 20 |  |
| Qualifier1 | D_6_SA | / | 139.1 | 96.1 | 20 | 9.327 |
| **Quantifier** |  |  | 139.1 | 81.1 | 16 |  |
| Qualifier2 | Jasmonic acid (JA) |  | 224.2 | 151 | 4 | 10.192 |
| Qualifier1 |  |  | 151.1 | 133.1 | 4 |  |
| **Quantifier** |  |  | 151.1 | 93.1 | 4 |  |
| **Quantifier** | Jasmonic acid-Isoleucine (Jaile) |  | 151.1 | 93 | 4 | 10.376 |
| Qualifier1 |  |  | 224.1 | 150.8 | 4 |  |
| Qualifier1 | H_2_JA | / | 153.1 | 83 | 20 | 10.203 |
| **Quantifier** |  |  | 153.1 | 97.1 | 8 |  |
| **Quantifier** | Indole-3-acetic acid (IAA) |  | 130.1 | 103.1 | 20 | 11.256 |
| Qualifier |  |  | 189.1 | 130 | 20 |  |
| **Quantifier** | D_2_IAA | / | 191.1 | 132 | 20 | 11.247 |
| **Quantifier** | Indole-3-carboxylic acid (ICA) |  | 144 | 89.1 | 28 | 11.494 |
| Qualifier2 |  |  | 175 | 89 | 28 |  |
| Qualifier1 |  |  | 144 | 116 | 20 |  |
| **Quantifier** | Indole-3-butyric acid (IBA) |  | 130.1 | 103.1 | 20 | 12.291 |
| Qualifier1 |  |  | 217.1 | 130 | 20 |  |
| Qualifier2 |  |  | 217.7 | 142.9 | 20 |  |
| **Quantifier** | 2-cis-4-trans Abscisic acid (ABA) |  | 190.1 | 162 | 8 | 12.55 |
| Qualifier1 |  |  | 190.1 | 91 | 28 |  |
| Qualifier2 |  |  | 162.1 | 134 | 4 |  |
| **Quantifier** | D_6_ABA | / | 194.1 | 165.8 | 8 | 12.526 |
| Qualifier3 | 12-oxophytodienoic acid (OPDA) |  | 238.2 | 148.9 | 20 | 13.479 |
| Qualifier2 |  |  | 163.1 | 121.1 | 10 |  |
| **Quantifier** |  |  | 121.1 | 77 | 20 |  |
| Qualifier4 |  |  | 238.2 | 206 | 4 |  |
| Qualifier1 |  |  | 121.1 | 93 | 8 |  |
|  |  |  |  |  |  |  |

1. Chromatogram for the phytohormones and organic acids


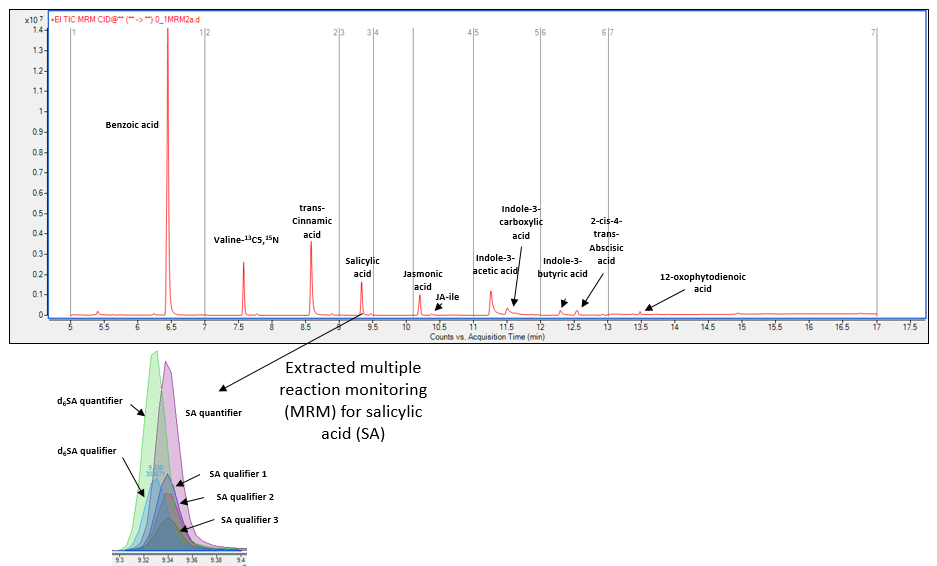

Supplement: Supplementary file 1 — Supplementary material 1 (DOCX 103 kb) [file 11306_2019_1606_MOESM1_ESM.docx]
