# Supplementary material for: Phenotyping reproductive stage chilling and frost tolerance in wheat using targeted metabolome and lipidome profiling
Source: Metabolomics. 2019 Oct 20;15(11):144. doi: 10.1007/s11306-019-1606-2 (PMC6800866; doi:10.1007/s11306-019-1606-2)
Supplement: Supplementary file 2 — Supplementary material 2 (DOCX 2792 kb) [file 11306_2019_1606_MOESM2_ESM.docx]

**Supplementary Fig. S1**


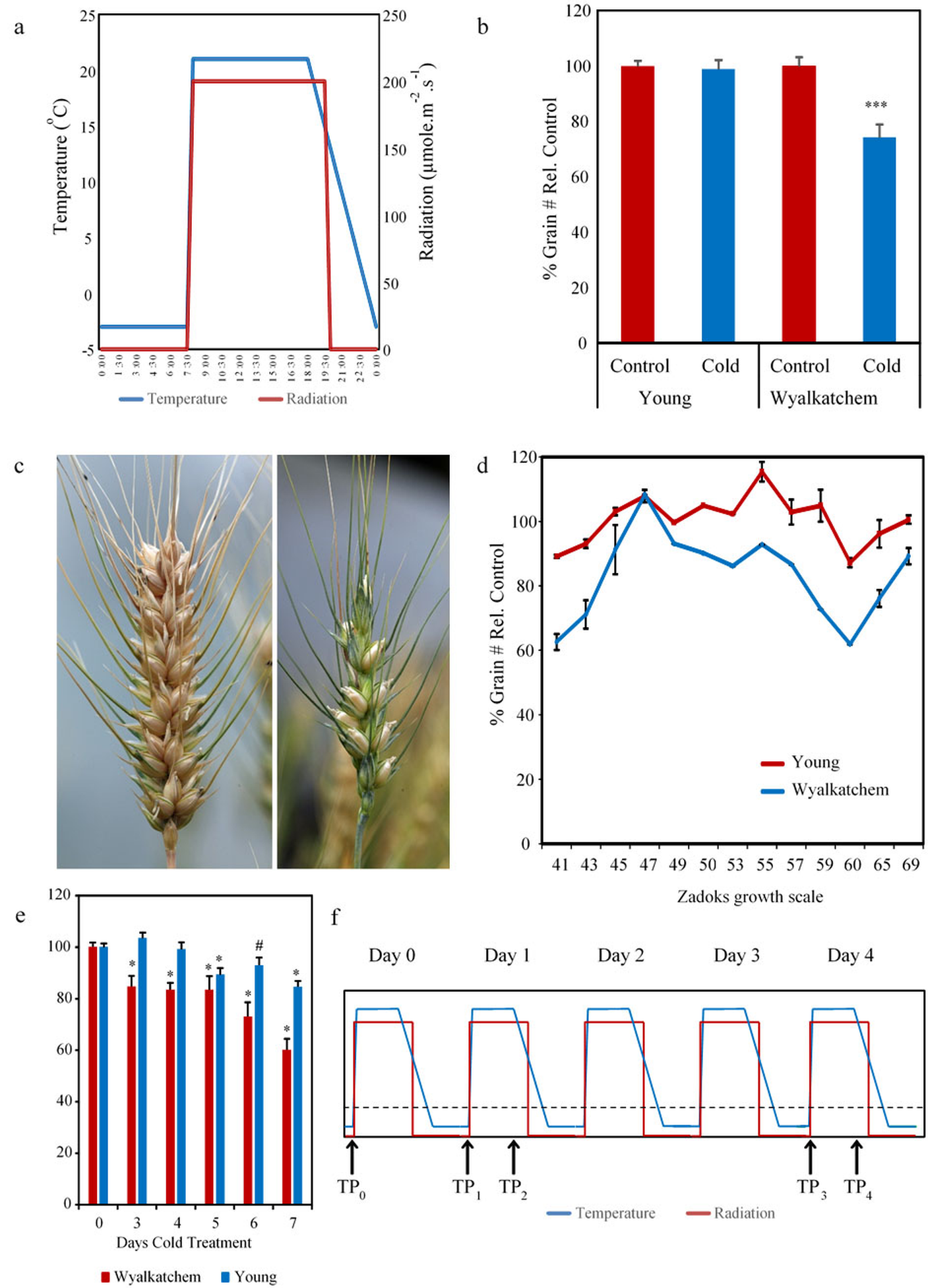


**Fig. S1** Schematic of the controlled environment chilling tolerance assay. **a.** Profile of temperature and lighting used in the controlled environment cabinets. **b.** Average spike grain number relative to unstressed control plants after a four-day chilling treatment at the YM stage in Young and Wyalkatchem. **c.** Larger grain size observed in fertile Wyalkatchem spikelets where spike grain number was reduced by four-day chilling treatment. **d.** Effect of cold treatment during different stages of flowering. Zadoks growth scale was used to determine flowering stages for the two cultivars Young and Wyalkatchem. A standard four-day cold treatment was applied. **e.** Time course experiment for cold treatment over seven days showing the effect of longer cold treatments on spike fertility for Young and Wyalkatchem. **f.** Graphic representation for the experimental design used for leaf harvesting and metabolite analysis

**Supplementary Fig. S2**


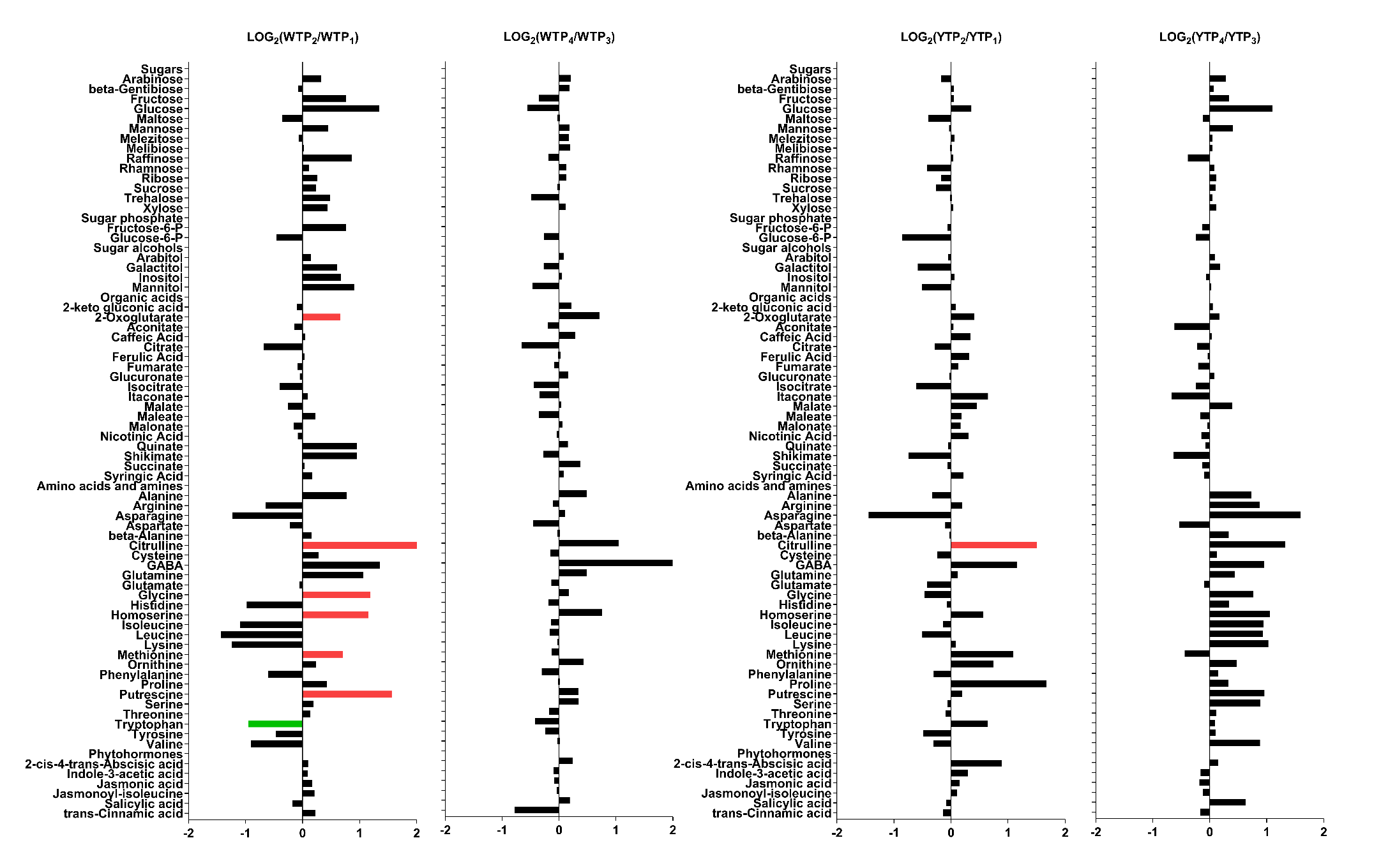


**Fig. S2** Log_2_-fold changes of primary metabolite recoveries in the flag leaves of the cold-sensitive Wyalkatchem (W) and cold-tolerant Young (Y) after one night (TP_2_ vs TP_1_) and prolonged (TP_4_ vs TP_3_) of cold treatment. Fold changes were calculated by dividing the concentration of the variety at a time point (eg. TP_2_) to the concentration of the variety at the previous time point (eg. TP_1_), then Log_2_-transformed. Significance of difference was determined by Benjamini and Hochberg method (Benjamini and Hochberg, 1995) with false discovery rate (FDR)-adjusted *p*-value of 0.05 as the cut-off. Green = significant decrease; Red = significant increase. There were four biological replicates (n=4) for all the measured metabolites, except phytohormones (n = 3).

**Supplementary Fig. S3**


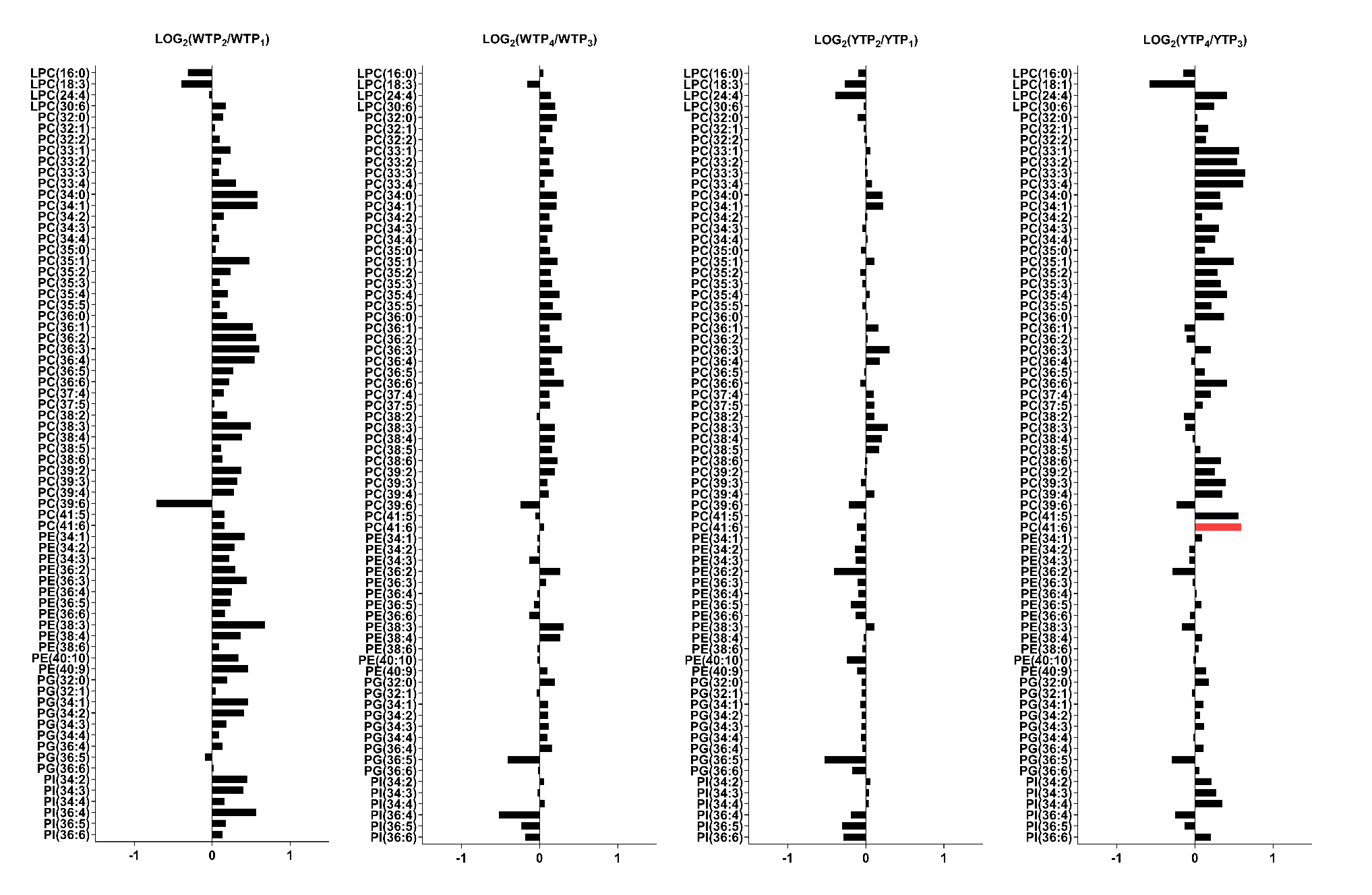


**Fig. S3** Log_2_-fold changes of phospholipid recoveries in the flag leaves of the cold-sensitive Wyalkatchem (W) and cold-tolerant Young (Y) after one night (TP_2_ vs TP_1_) and prolonged (TP_4_ vs TP_3_) of cold treatment. Fold changes were calculated by dividing the normalized response of the variety at a time point (eg. TP_2_) to the normalized response of that variety at the previous time point (eg. TP_1_), then Log_2_-transformed. Statistical method and cut-off are as stated in Fig. S2. Green = significant decrease; Red = significant increase. They were four biological replicates (n=4) for all the measured lipids.
